# The microbiota and T cells non-genetically modulate inherited phenotypes transgenerationally

**DOI:** 10.1016/j.celrep.2024.114029

**Published:** 2024-04-04

**Authors:** Jordan C. Harris, Natalie A. Trigg, Bruktawit Goshu, Yuichi Yokoyama, Lenka Dohnalová, Ellen K. White, Adele Harman, Sofía M. Murga-Garrido, Jamie Ting-Chun Pan, Preeti Bhanap, Christoph A. Thaiss, Elizabeth A. Grice, Colin C. Conine, Taku Kambayashi

**Affiliations:** 1 Department of Pathology and Laboratory Medicine, Perelman School of Medicine at the University of Pennsylvania, Philadelphia, PA 19104, USA; 2 Department of Dermatology, Perelman School of Medicine at the University of Pennsylvania, Philadelphia, PA 19104, USA; 3 Division of Neonatology, Children’s Hospital of Philadelphia, Philadelphia, PA 19104, USA; 4 Departments of Genetics and Pediatrics - Penn Epigenetics Institute, Institute of Regenerative Medicine, and Center for Research on Reproduction and Women’s Health, University of Pennsylvania Perelman School of Medicine, Philadelphia, PA 19104, USA; 5 Department of Microbiology, Perelman School of Medicine at the University of Pennsylvania, Philadelphia, PA 19104, USA; 6 Transgenic Core, Children’s Hospital of Philadelphia, Philadelphia, PA 19104, USA; 7 These authors contributed equally; 8 Lead contact

## Abstract

The host-microbiota relationship has evolved to shape mammalian physiology, including immunity, metabolism, and development. Germ-free models are widely used to study microbial effects on host processes such as immunity. Here, we find that both germ-free and T cell-deficient mice exhibit a robust sebum secretion defect persisting across multiple generations despite microbial colonization and T cell repletion. These phenotypes are inherited by progeny conceived during *in vitro* fertilization using germ-free sperm and eggs, demonstrating that non-genetic information in the gametes is required for microbial-dependent phenotypic transmission. Accordingly, gene expression in early embryos derived from gametes from germ-free or T cell-deficient mice is strikingly and similarly altered. Our findings demonstrate that microbial- and immune-dependent regulation of non-genetic information in the gametes can transmit inherited phenotypes transgenerationally in mice. This mechanism could rapidly generate phenotypic diversity to enhance host adaptation to environmental perturbations.

## In brief

Harris et al. describe phenotypic abnormalities in germ-free and T cell-deficient mice that are not acutely correctable and are non-genetically transmitted to progeny. The parental microbe and immune environment impact gametes to alter early embryonic gene expression, thereby influencing barrier and metabolic tissue of progeny through transgenerational non-genetic inheritance.

## Graphical abstract

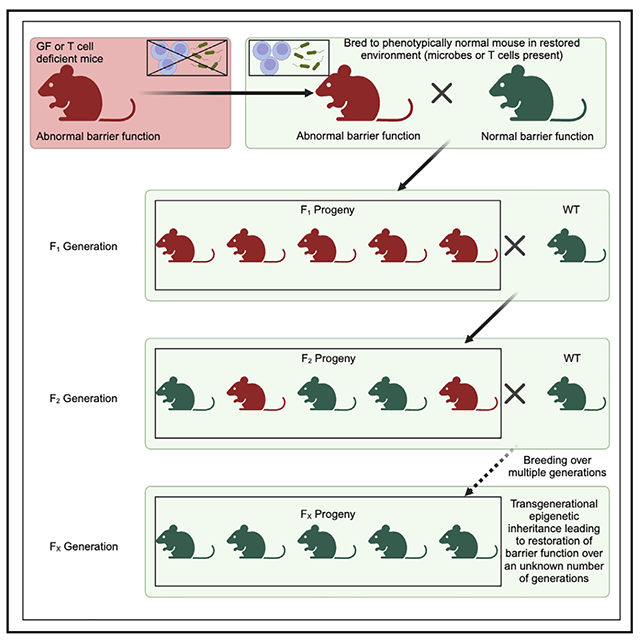

## INTRODUCTION

Barrier sites including skin, gut, and lung are responsible for responding to a wide variety of environmental perturbations, including exposure to pathogens, physical disruption, and altered nutrient homeostasis.^1–6^ The ability of these tissues to adapt to changing environments is a key component of organismal viability. In the long term, natural selection and evolution allow for optimal adaptation to many of these environmental shifts, while more severe and abrupt changes, such as infection, garner more acute responses. Just as a stratified, keratinized layer of skin has evolved over long periods to provide a permanent external barrier, the presence of skin commensal bacteria and the mechanisms by which they prevent pathogenic invasion allows for a more short-term form of cutaneous defense.^7,8^

Phenotypic diversity induced by genetic mutations, which are randomly introduced and accrue slowly over time, may not efficiently allow acute adaptation to changing environmental conditions. In contrast, environmentally regulated non-genetic (i.e., cross-generational information transfer not explainable by genetic inheritance) gene regulation could serve as a more rapid adaptive mechanism to induce phenotypic changes. Organisms may “fine-tune” phenotypes in response to environmental factors, which in some cases can be transmitted to their offspring. Indeed, recent work in *C. elegans* demonstrated the transmission of environmentally regulated, persistent phenotypes across generations even in the absence of the initial environmental perturbation.^9,10^ Although it has been recently established that mammalian phenotypes affected by parental diet can be transmitted to F_1_ progeny intergenerationally,^11,12^ whether non-genetic or epigenetically inherited information regulated by the environment can transmit phenotypes transgenerationally, i.e., to the F_2_ generation and beyond, remains controversial. Most skepticism toward transgenerational epigenetic inheritance occurring in mammals emerges from a dearth of a mechanistic explanation. Mechanistic studies require robust readouts, but while examples of parental exposures to chemicals have demonstrated transgenerationally inherited phenotypes in rodents,^13–15^ the few examples of transgenerational epigenetic inheritance of environmentally modulated phenotypes, such as stress and diet, are more variable.^16–18^ Thus, a robust and reliable readout to study these inheritance mechanisms will advance the field by allowing mechanistic transgenerational studies.

In *C. elegans*, several described examples of transgenerational epigenetic inheritance occur by environmental stimuli that are initiated at the gut barrier, which regulate epigenetic information in the germline to transmit phenotypes to subsequent generations of progeny. However, in mammals, it has yet to be examined whether the environment can modulate information communicated between the germline and barrier surfaces and, further, whether this communication can modulate offspring phenotypes. One potential candidate that could allow transmission of environmental information from barrier surfaces to the host is the microbiome. Host microbiota are exquisitely sensitive to large environmental or host-specific shifts including changes to diet, pollution, immune cell populations, and stress.^19–22^ Importantly, the microbiota is in direct communication with both the external world and host tissue, which makes it optimally poised to rapidly respond to the environment and promote adaptation. Interestingly, germ-free (GF) mice display some phenotypic changes that are not restorable by acute microbial colonization, suggesting that the change is not caused by an acute loss of microbes in the host.^23^ Thus, although not reported thus far, it is possible that certain phenotypes of GF mice persist across generations despite microbial colonization.

We recently described a cutaneous immune-sebum circuit whereby thymic stromal lymphopoietin (TSLP)-stimulated T cells can influence the ability of sebaceous glands (SGs) to secrete sebum, an oily substance that promotes skin hydration, acidification, and anti-microbial defense.^24–26^ Since T cells can be activated by microbial antigens and TSLP can be released from skin keratinocytes with stimulation by microbial products,^27–29^ we hypothesized that the skin microbiota could trigger T cell activation and TSLP expression to induce sebum secretion, which would in turn control skin commensals. This feedback mechanism could maintain a delicately balanced skin ecosystem, which is essential for optimal barrier function.^23,30^ In this study, we found that skin microbiota does indeed control sebum secretion, albeit not in an acute manner as originally hypothesized. Instead, we found that commensal microbes influence SG function, as well as the transcriptional profiles in multiple organs by transgenerational non-genetic inheritance. Further, we find that T cells additionally regulate analogous transgenerationally inherited phenotypes, including defective sebum secretion. Both the microbiota and T cells strikingly influence gene expression of early embryos, which has the potential to modulate development, thereby programming non-genetically inherited phenotypes. Our results reveal that the microbiome and immune system control epigenetic information in the gametes to modulate the phenotypes of succeeding generations of progeny.

## RESULTS

### GF mice possess a dysfunctional cutaneous immune-sebum circuit

**Figure 1.**
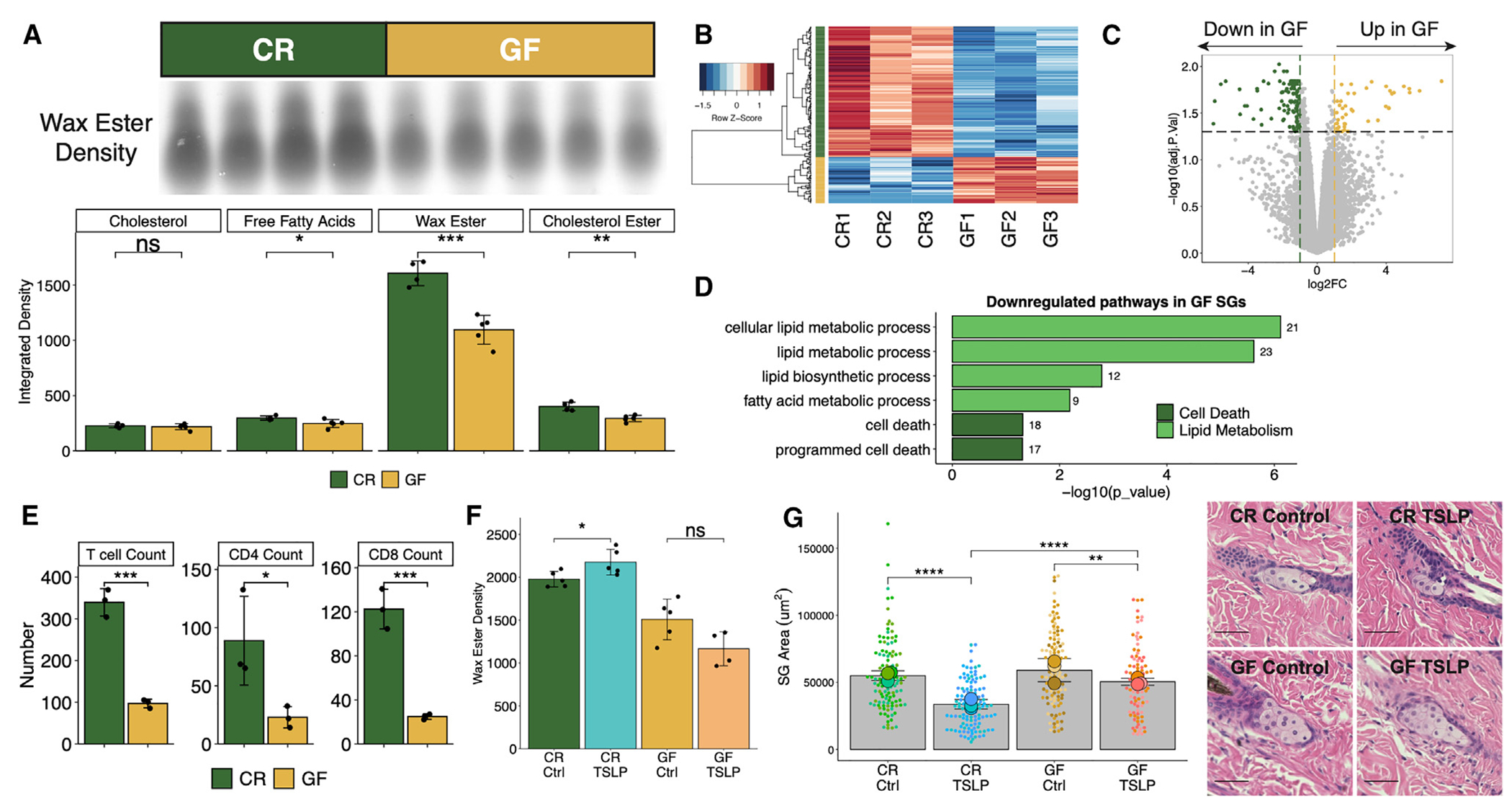
GF mice display a defective immune-sebum circuit (A) Wax ester intensity and hair sebum lipid quantification by thin-layer chromatography (TLC) (*n* = 4 or 5 mice/group). (B) Heatmap of DEGs quantitated from bulk mRNA-seq of LCM-isolated GF or CR mouse SGs (*n* = 3 mice/group). (C) Volcano plot highlighting SG genes upregulated (45 genes) and downregulated (127 genes) in GF mice. (D) Selected GO terms downregulated in GF SGs. Number of genes within the dataset within each term is listed beside the bar. (E) Number of skin T cells (*n* = 3 mice/group). (F and G) CR or GF mice treated intravenously with one dose of 5 × 10^10^ genome copies of control- or TSLP-adeno-associated virus (AAV) for 14 days (*n* = 4 or 5 mice/group). (F) TLC quantification of wax ester from hair (*n* = 4 or 5 mice/group). (G) SG area with representative H&E images (scale bars, 100 μm; *n* = 3 mice/group, *n* = 27–53 SGs/mouse). Sequencing experiments were performed once. All other experiments were performed 2–5 times. ns, not significant, *p < 0.05, **p < 0.01, ***p < 0.001, and ****p < 0.0001 by Student’s t test. Data are shown as mean ± SD. See also Figure S1.

We have previously shown that GF mice display abnormal epidermal structure and barrier function.^23,31^ To determine if the skin barrier defect carries to SG function, we examined an RNA sequencing (RNA-seq) dataset generated by our lab (GEO: GSE162925) and found that GF epidermis showed reduced expression of lipid metabolism and anti-microbial peptide genes, processes that are both important in SG biology (Figure S1A). To test SG function and measure sebum secretion, a standardized area of fur was shaved from conventionally raised (CR) control and GF mice, and fur lipids were then extracted and separated via thin-layer chromatography (Figure S1B). Consistent with the skin transcriptomic findings, the amount of sebum present on the fur of GF mice was significantly reduced compared to CR mice (Figure 1A). To interrogate whether the SGs themselves were defective, we isolated SGs from formalin-fixed, paraffin-embedded CR and GF skin using laser capture microdissection (LCM; Figure S1C),^32^ extracted RNA, and performed RNA-seq to identify any transcriptomic abnormalities present. This investigation revealed a distinct transcriptional signature in SGs of GF mice (Figures 1B and S1D), with 45 genes significantly upregulated and 127 genes significantly downregulated in GF SGs (Figure 1C). Gene Ontology (GO) and gene set enrichment analysis (GSEA) revealed that GF SGs displayed downregulation of lipid metabolism and cell death pathways, processes important in SG lipogenesis and holocrine (cell-death-mediated) sebum secretion (Figures 1D and S1E).^33,34^

We previously defined a cutaneous immune-sebum circuit whereby TSLP-stimulated T cells control SG function.^24^ To test whether the sebum secretion defect in GF mice was related to the immune-sebum circuit, we examined T cell numbers and expression of *Tslp* in skin of GF mice. We found that GF skin exhibited significantly reduced T cell numbers, as well as a trend toward reduced TSLP expression, compared to CR skin (Figures 1E and S1F). As we have previously shown that TSLP overexpression leads to sebum hypersecretion and SG size reduction (due to increased holocrine secretion),^24^ we tested whether TSLP overexpression could restore sebum secretion in GF mice. GF mice treated with TSLP showed unaltered sebum secretion and a less profound change in SG size compared to TSLP-treated CR mice (Figures 1F and 1G). Together, these data suggest that GF mice harbor a defect in homeostatic sebum secretion that cannot be overcome by TSLP overexpression.

### Many cutaneous GF phenotypes persist despite microbial colonization

**Figure 2.**
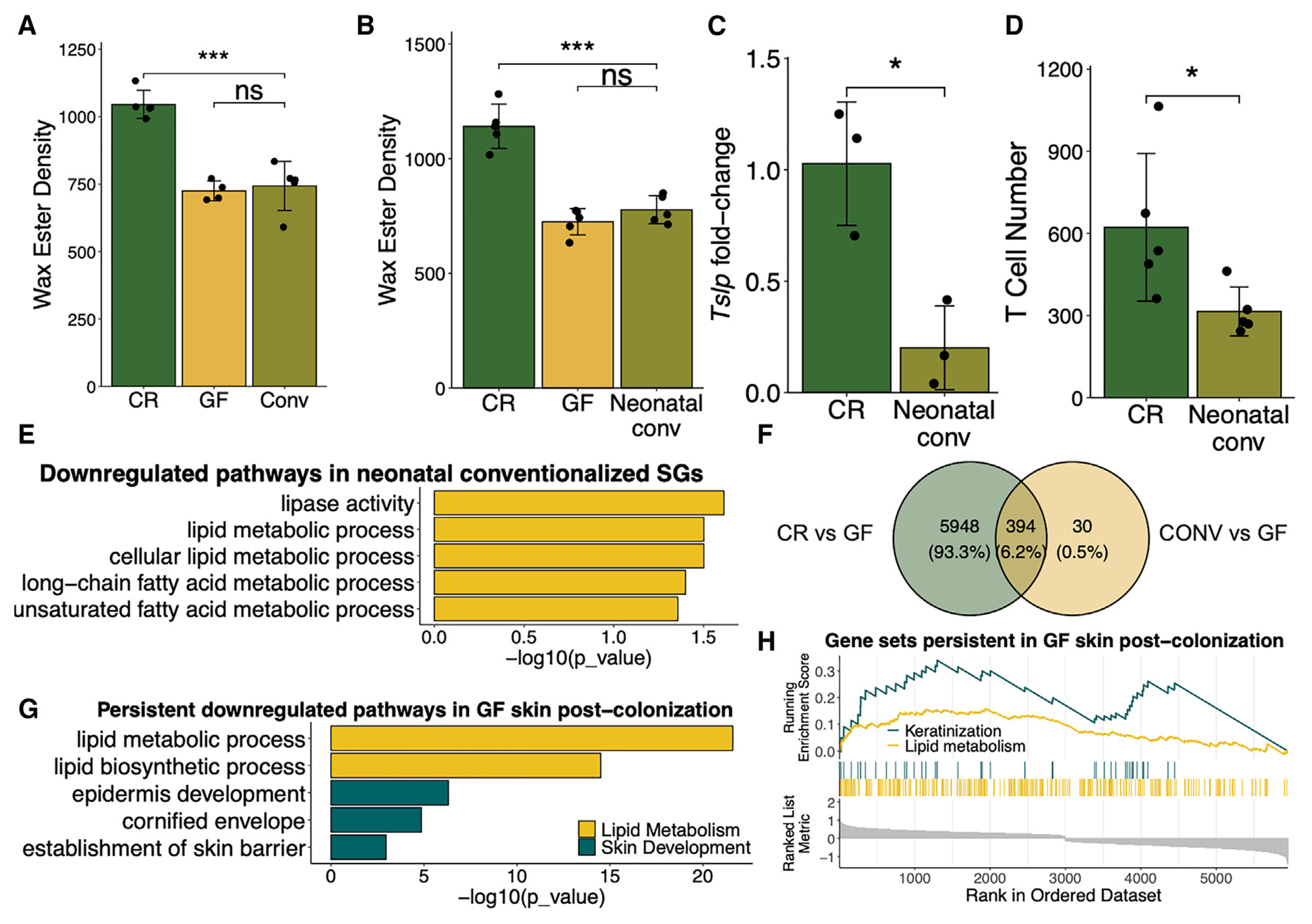
GF cutaneous phenotypes display inherent resistance to rescue via microbial conventionalization (A and B) TLC quantification of hair wax esters from CR, GF, and (A) 8 week post-conventionalized (CONV) adult GF mice (*n* = 4 or 5 mice/group) or (B) GF mice CONV from birth (*n* = 4 or 5 mice/group). (C) Skin mRNA expression of *Tslp* in mice CONV from birth compared to controls (*n* = 3 mice/group, qPCR normalized to *Gapdh* expression). (D) Number of skin T cells by flow cytometry in mice CONV from birth compared to controls (*n* = 3 mice/group). (E) Selected downregulated lipid-related GO terms as discovered by RNA-seq of control or neonatally CONV SGs (*n* = 2 or 3 mice/group). (F–H) Data from bulk RNA-seq derived from CR, GF, and CONV adult murine epidermis (*n* = 8 mice/group). (F) Distinct and overlapping DEGs from CR or CONV compared to GF epidermis. (G) Selected downregulated GO pathways, which persist in GF mice post-colonization. (H) GSEA plot displaying persistent downregulated pathways in GF epidermis post-colonization using Benjamini-Hochberg (BH)-adjusted *P* value < 0.05. Genes in GSEA plot are shown in ranked order by running enrichment scores. Sequencing experiment was performed once. All other experiments were performed 2–3 times. ns, not significant, *p < 0.05, **p < 0.01, and ***p < 0.001 by Student’s t test. Data are shown as mean ± SD. See also Figure S2.

To begin to understand how the absence of microbes in GF mice affects the immune-sebum circuit, we attempted to rescue the sebum secretion defect in GF mice. Since many phenotypic alterations in GF mice can be corrected by microbial colonization, 8-week-old adult GF mice were transferred to our conventional facility and housed in cages with added bedding and other cage materials from CR mice, as previously described (Figure S2A).^23^ After 8 weeks of colonization, we still found that sebum secretion remained defective in the transferred adult GF mice (Figure 2A) despite adequate restoration of skin commensals (Figure S2B). Since colonization during the neonatal period is critical for rescuing certain phenotypes in adult GF mice,^35–37^ we tested if the sebum secretion defect in GF mice could only be corrected if microbially colonized from birth. We thus conventionally colonized pregnant GF dames and measured adult sebum secretion in the pups that were colonized from birth (Figure S2C). Surprisingly, conventionalization of pregnant GF dames still gave rise to adult progeny with a sebum secretion defect (Figure 2B). These data suggest that the GF sebum secretion defect is more complex than simply the presence or absence of microbes.

To determine if the persistence of GF sebum secretion after conventionalization extended to other GF immune-sebum circuit defects (such as those observed in Figures 1D, 1E, and S1D), we examined T cell numbers and *Tslp* expression in the skin of mice born from a GF dame conventionalized (CONV) during pregnancy (Figure S2C). Similar to the sebum secretion defect, reduced cutaneous *Tslp* expression and T cell numbers also persisted in adult GF mice CONV from birth (Figures 2C and 2D). Further, to corroborate the persistent defective sebum secretion findings, we performed RNA-seq of LCM-isolated SGs from the CONV GF mice compared to CR control mice. Lipid-metabolism-related pathways that were downregulated in the SGs of GF mice were also downregulated in the GF mice CONV from birth (Figure 2E). These data confirm that not only sebum secretion but also other defects in the immune-sebum pathway remain defective in CONV GF mice.

Lastly, to examine the persistence of GF phenotypes beyond the immune-sebum circuit, we used a full epidermis bulk mRNA-seq gene expression dataset derived from CR, GF, and CONV adult mice^23^ (GEO: GSE162925) to interrogate the propensity of genes to continue displaying altered expression after microbial conventionalization. In our previous work, we found a total of 6,396 differentially expressed genes (DEGs) in the epidermis of CR compared to GF mice and only 427 DEGs in the skin of CONV compared to GF mice.^23^ Thus, the vast majority of DEGs (~6,000 DEGs) seen in the epidermis of GF compared to CR mice are persistently altered in GF mice and unaffected by microbial colonization (Figure 2F). We extracted this list of genes that were not rescued by microbial colonization and performed GO analysis and GSEA on the subset of genes that were persistently downregulated in the epidermis of GF mice. We found that there were many processes related to skin barrier development that remained downregulated in GF mice after colonization, including those related to cornification, keratinization, and epidermal development (Figures 2G and 2H). Additionally, we found that many downregulated lipid metabolism terms in GF skin remain downregulated after colonization (Figures 2G and 2H). Overall, these data suggest that many GF phenotypes persist despite colonization of GF mice from birth, raising the possibility that there may be an inherited factor leading to persistent phenotypic changes.

### The reduced sebum secretion phenotype of GF mice is transmitted to progeny transgenerationally

**Figure 3.**
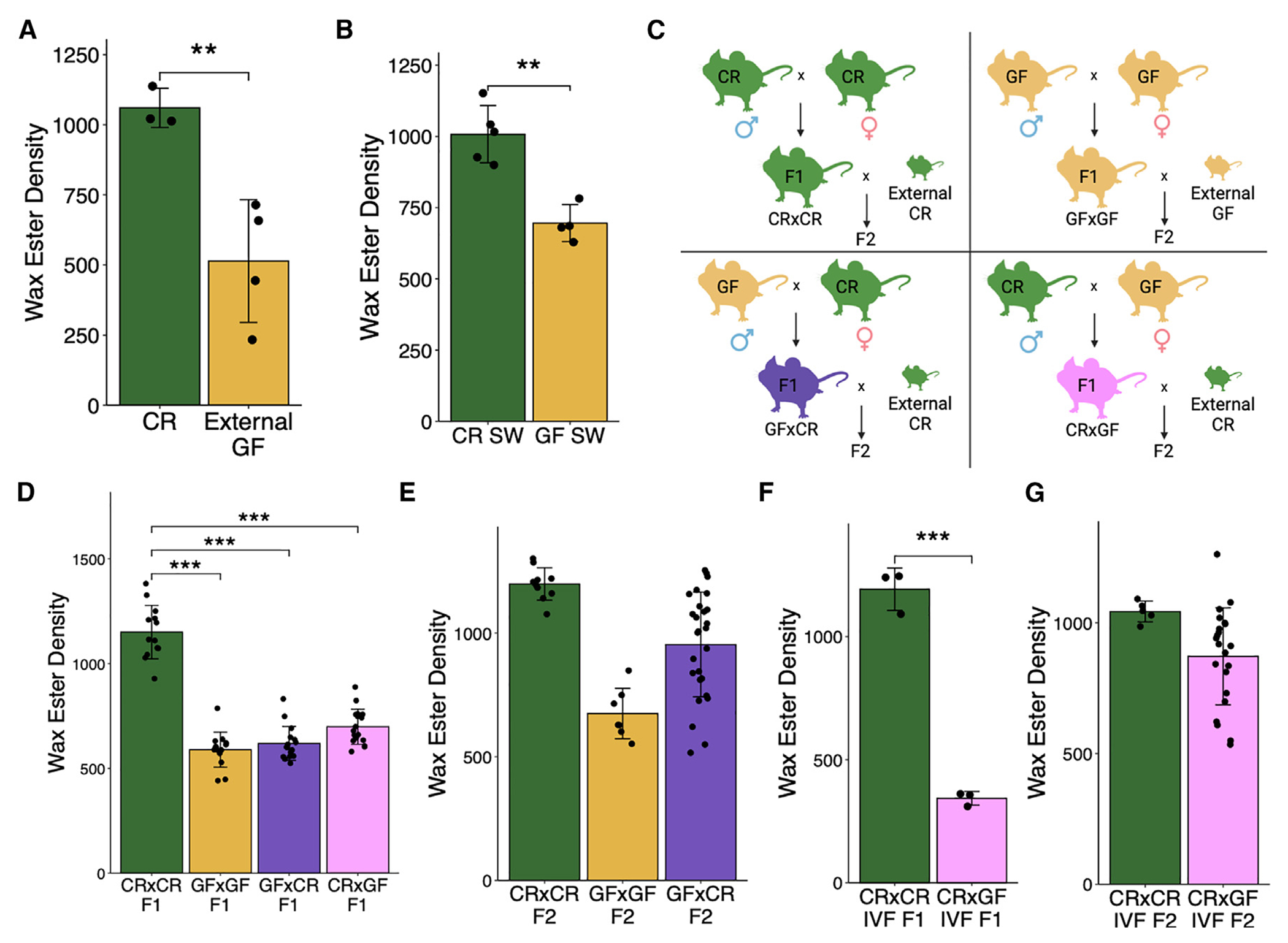
Sebum phenotypes are transmitted to progeny transgenerationally from GF mice (A and B) TLC quantification of hair wax esters from (A) CR or GF mice from the UNC gnotobiotic core (*n* = 3 or 4 mice/group) and (B) CR or GF Swiss-Webster mice (*n* = 4 or 5 mice/group). (C) Breeding scheme for transgenerational experiments. (D–G) TLC quantification of hair wax esters from the progeny of combinatorial CR and GF natural breeding in the (D) F_1_ (*n* = 13 to 17 mice/group, two F_0_ breeding pairs/group) and (E) F_2_ (*n* = 7 to 27 mice/group, two F_1_ breeding pairs/group) generations and mice derived from IVF and the resulting (F) F_1_ (*n* = 3 mice/group) and (G) F_2_ (*n* = 6 or 22 mice/group) generations. All experiments performed 2–3 times. **p < 0.01, ***p < 0.001 by Student’s *t* test. Data are shown as mean ± SD. See also Figure S3.

One possibility for why SG activity was not restorable in the colonized offspring of GF mice (C57BL/6 strain) in our gnotobiotic facility (University of Pennsylvania) could be that this colony had allopatrically acquired a genetic mutation that was responsible for preventing physiologic sebum secretion through genetic drift.^38^ To test this, we examined sebum secretion in GF C57BL/6 mice from another gnotobiotic facility (University of North Carolina [UNC]) and in another GF strain (Swiss-Webster) from our facility. Both the GF C57BL/6 strain from UNC and the GF Swiss-Webster mice displayed a secretion sebum defect similar to GF C57BL/6 mice from our colony (Figures 3A and 3B). Together, these data argued against a randomly acquired genetic mutation in GF mice as the cause of the sebum secretion defect.

To test if the sebum secretion defect was heritable, we bred CR mice with GF mice in a conventional animal facility in all four combinations: CR male × CR female (CR×CR), GF male × CR female (GF×CR), CR male × GF female (CR×GF), and GF male × GF female (GF×GF) (Figure 3C). The F_1_ progeny with at least one GF parent displayed a sebum secretion defect comparable to that of parental GF mice (Figure 3D), suggesting that the GF sebum secretion phenotype is dominantly inherited (100% of mice in all groups with a GF parent inherited the defective phenotype). This was despite similar skin and gut microbiota as measured by culturable colony-forming units and alpha diversity metrics via 16S rRNA gene amplicon sequencing (Figures S3A and S3B). In some experiments, a small minority (16 of 148) of F_1_ mice with a GF parent displayed normal sebum secretion. To test whether the phenotype persisted in the F_2_ generation, CR×CR and GF×CR F_1_ female mice were bred with new CR male mice, while female mice from the GF×GF group were bred with new GF male mice as a negative control (Figure 3C). Approximately half of the GF×CR group in the F_2_ generation remained defective, portraying a stochastic “restored-or-defective” phenotype of sebum secretion despite being from the same litter (Figure 3E, ~59% of the GF×CR group and 100% of the GF×GF group inherited the defective phenotype). This heritable sebum secretion defect is unlikely a result of transmission of an intrinsic SG defect, as we measured sebum secretion of F_1_ heterozygous mice derived from breeding *Scd1*^−/−^ mice (which develop atrophic and dysfunctional SGs^39^) with wild-type (WT) mice. There was not a deficiency of sebum secretion in F_1_ heterozygous progeny (Figure S3C), supporting our hypothesis that this defect is related to the ancestral lack of a microbiome. Overall, this pattern of inheritance suggested that the sebum secretion defect was transgenerationally inherited, as both males and non-pregnant females transmit the phenotype to the F_2_ generation.

As an alternative approach to confirm the transgenerational non-genetic inheritance pattern, we carried out a similar breeding strategy but bred littermates of each generation and measured sebum secretion in the F_1_, F_2_, and F_3_ generations (Figure S3D). Here, we find similarly that F_1_ mice with at least one GF parent retain a sebum secretion defect despite similar microbial colonization (Figure S3E, 100% of mice in all groups with a GF parent inherited the defective phenotype). Males and females from the F_1_ generation were then bred together, resulting in an F_2_ generation, which showed a similar pattern of stochasticity in the GF×CR group with half of the mice displaying a CR sebum secretion phenotype and half displaying a GF sebum secretion phenotype (Figure S3F, 50% of the GF×CR group and 100% of the CR×GF and GF×GF groups inherited the defective phenotype). Finally, F_2_ males and female mice were bred, generating an F_3_ cohort, of which all groups originating from a GF F_0_ ancestor had a subset of offspring with defective sebum secretion and portrayed stochasticity seen in the GF–GF and CR×GF groups, though the effect size was reduced compared to previous experiments (Figure S3G, 50% of the GF×GF and CR×GF groups and 100% of the GF×CR group inherited the defective phenotype).

Finally, to ensure that the phenotype can be transmitted by the gametes of GF mice in the absence of potentially confounding environmental factors, such as microbiome transfer or maternal care, *in vitro* fertilization (IVF) with subsequent implantation into surrogate mothers was performed. Similar to results obtained with natural breeding, sebum secretion was defective in F_1_ progeny when eggs or sperm were of GF origin (Figure 3F, 100% of mice in the CR×GF IVF group inherited the defective phenotype). In the F_2_ generation, the sebum secretion defect persisted in approximately one-third of F_2_ offspring of CR×GF IVF mice mated to CR mice (Figure 3G, ~73% of mice in the CR×GF IVF group inherited the defective phenotype). Thus, we have demonstrated in two natural breeding schemes as well as in IVF that the sebum secretion defect of GF mice is transmitted to at least the F_2_ generation. These results demonstrate that the sebum secretion phenotype of GF mice is transmitted transgenerationally after removal of the environmental perturbation (in this case, the lack of microbiota) but is restored sporadically over time.

### Transgenerational epigenetic inheritance from GF mice is not restricted to sebum secretion

**Figure 4.**
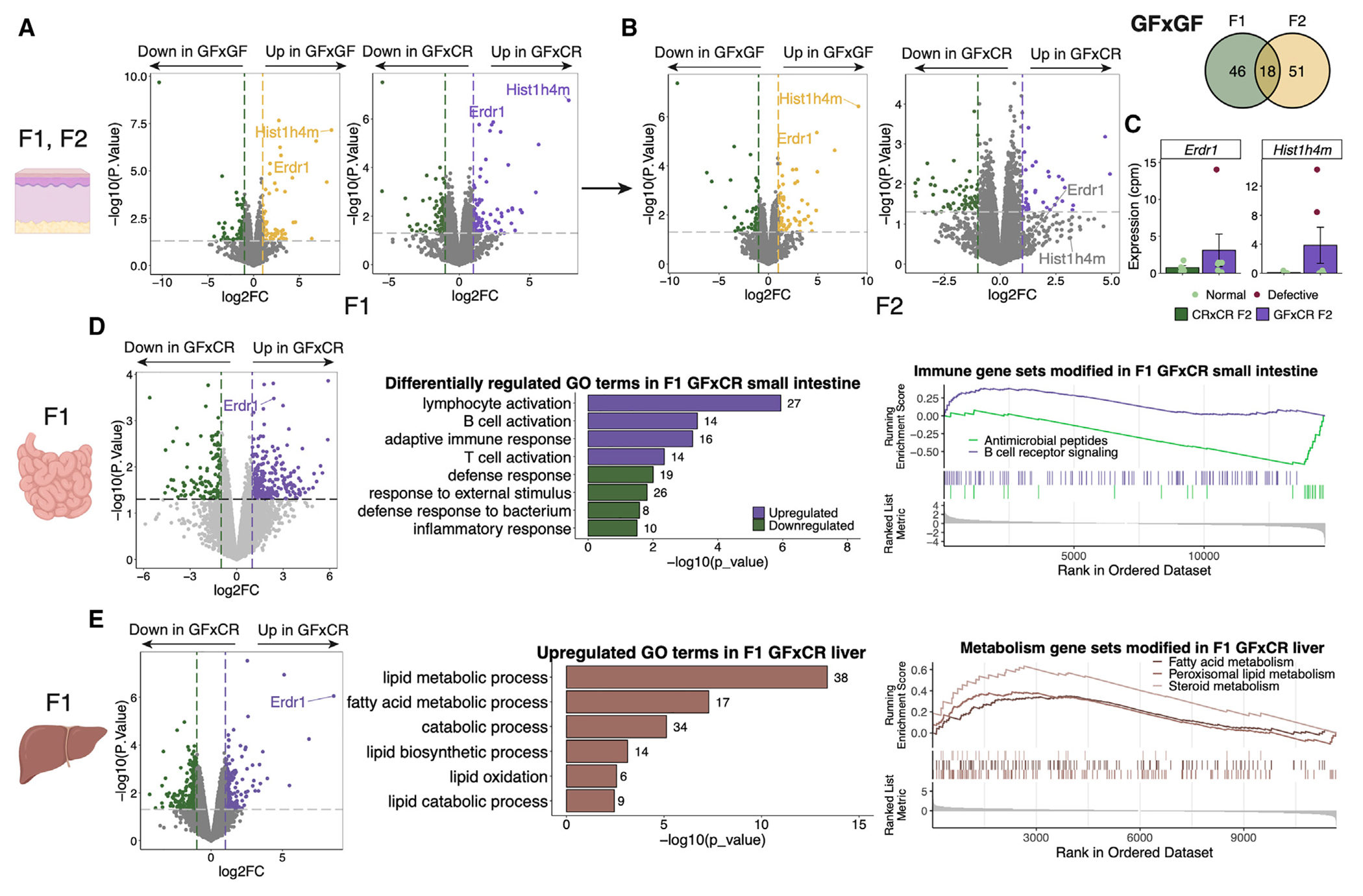
GF barrier and metabolic tissue display transgenerational transcriptional dysfunction (A–C) Gene expression by RNA-seq of F_1_ and F_2_ CR×CR, GF×GF, and GF×CR back skin (*n* = 3–6 mice/group). Volcano plots representing pairwise group comparisons of DEGs across (A) F_1_ and (B) F_2_ generations, highlighting two common genes and a Venn diagram highlighting all common genes between F_1_ and F_2_ GF×GF skin. (C) Counts per million of two F_1_ DEGs with bimodal expression in F_2_. (D) Gene expression by RNA-seq of F_1_ CR×CR and GF×CR small intestine (*n* = 3 or 4 mice/group) including DEGs, GO terms, and GSEA showing upregulated and downregulated pathways. (E) Gene expression by RNA-seq of F_1_ and CR×CR and GF×CR liver tissue (*n* = 3 or 4 mice/group) including DEGs, GO terms, and GSEA showing upregulated pathways. The number of genes within the dataset within each GO term is listed beside the bar. Genes in GSEA plot are shown in ranked order by running enrichment scores. Sequencing experiments were performed once. Data in bar plot are shown as mean ± SD. See also Figure S4.

We next tested whether the transgenerational epigenetic inheritance of phenotypes induced by the lack of microbes also extended to the regulation of other biological processes. We first performed RNA-seq of the skin of progeny of CR×CR, GF×GF, and GF×CR mice. Similar to the GF F_0_ mice, we found DEGs in the skin transcriptomic profile of GF×GF (139 DEGs) and GF×CR (174 DEGs) F_1_ mice compared to CR×CR F_1_ mice, suggesting that mice derived from even a single GF parent maintain altered cutaneous gene expression (Figures 4A and S4A). 18 DEGs from the F_1_ generation persisted to the F_2_ generation of the GF×GF group (Figures 4B and S4B). Some of these DEGs persisted but lost significance in the GF×CR F_2_ mice (Figures 4B and S4B) because the gene expression pattern in the F_2_ generation was bimodally distributed due to sporadic reversion of gene expression in a proportion of the progeny, mimicking the pattern of sebum inheritance. Two examples (*Erdr1* and *Hist1h4m*) are shown in the GF×CR F_2_ group, where the mice displayed a dichotomous “on-or-off” level of expression (Figure 4C). Interestingly, the recovery of gene expression levels of *Erdr1* and *Hist1h4m* in the GF×CR F_2_ group did not correlate with sebum secretion recovery in the same mice, suggesting that these genes may not be involved with transgenerational sebum secretion recovery but may be important in other non-genetically inherited biologic processes (Figure S4C).

It is known that GF mice have a dysregulated transcriptome in many tissues, including many barrier defense and metabolic sites.^31,40–46^ Thus, to determine whether the transgenerational epigenetic inheritance process in GF mice extended to transcriptomes of a broad range of body sites, we collected small intestine and liver from the progeny of CR×CR, GF×GF, and GF×CR mice. The small intestine is another barrier site and the liver is a metabolic tissue, and both have been shown to be transcriptionally dysregulated in GF mice.^42,44,46–48^ In the small intestine of GF×CR F_1_ mice, immune activity related to innate bacterial defense pathways was downregulated, while adaptive and lymphocytic immune pathways were upregulated, compared to CR×CR F_1_ mice (Figure 4D), suggesting an alteration in immune response to microbes, though these trends were not statistically significant with multiple comparison correction (Figure S4D). In the liver, GF×CR F_1_ tissue displayed a change in metabolic function, with both lipid biosynthetic and catabolic processes upregulated, suggesting differential processing of lipid species in the GF×CR F_1_ mice (Figures 4E and S4E). Taken together, these results suggest that the epigenetic inheritance pattern is not limited to SGs; multiple tissues in F_1_ mice derived from a GF parent are dysregulated even after colonization, suggesting that this process could represent a pervasive mechanism for controlling gene expression and phenotypes across generations of progeny.

### The microbiome of parents regulates early embryonic gene expression through gametes

**Figure 5.**
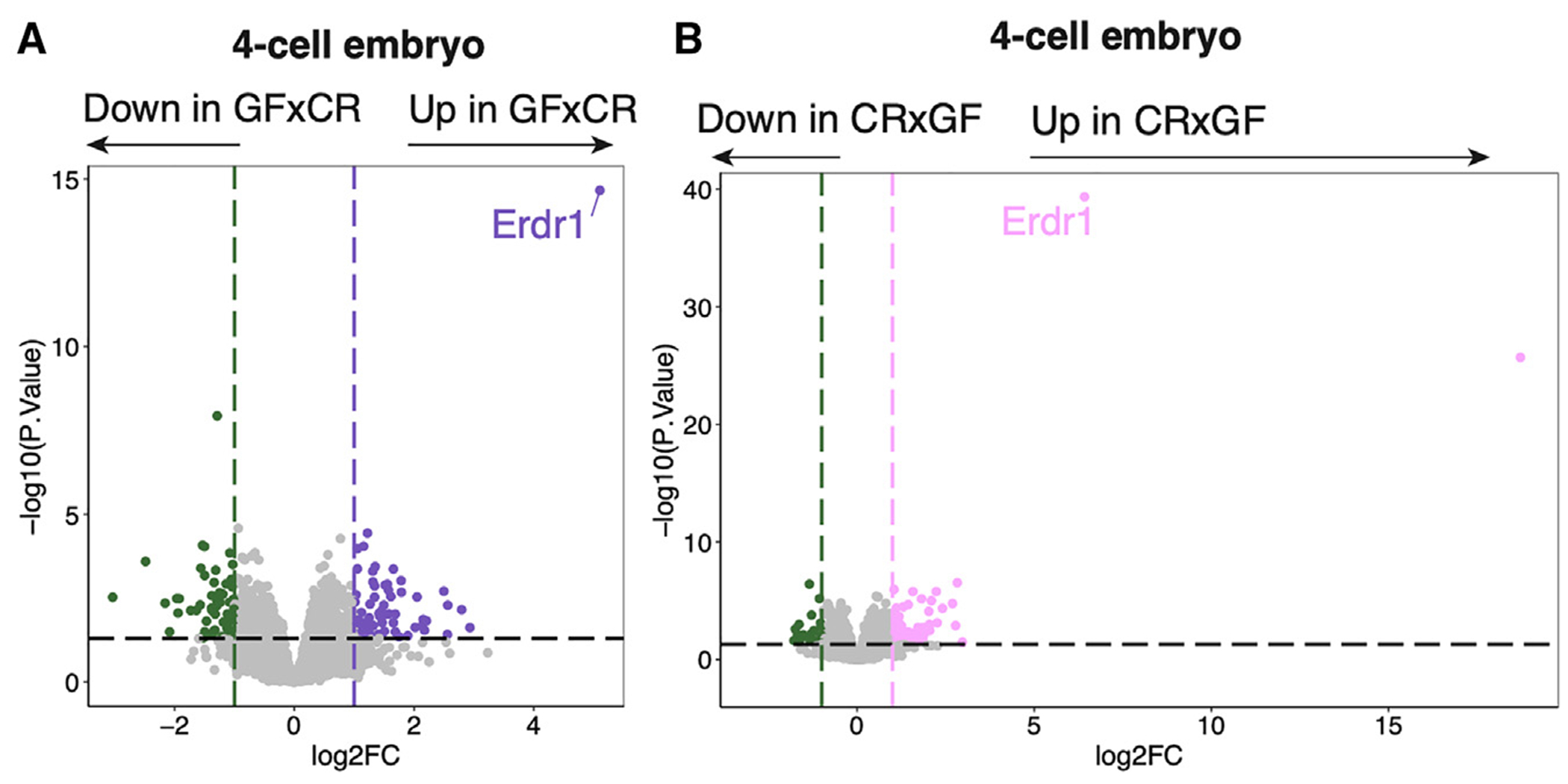
Early embryos from GF mice exhibit a distinct gene expression profile Gene expression by RNA-seq of CR×CR vs. GF×CR (A) and CR×CR vs. CR×GF (B) 4-cell embryos (*n* = 9–25 embryos/group, collected over three biological replicates of IVF). DEGs are defined as log_2_ fold change >1 or < −1, *p* < 0.05. Sequencing experiments were performed once. See also Figure S4.

To determine whether the observed transgenerational phenotypes and gene expression in adult tissue could be traced to early development, we determined whether IVF with GF gametes caused altered gene expression in the early embryo. Single-embryo mRNA-seq was performed at the 4-cell and morula stages after IVF with the sperm or eggs from GF or CR mice combined with the reciprocal gamete in GF or CR mice for a result of CR×CR, GF×CR, and CR×GF 4-cell embryos as well as CR×CR and GF×CR morulae. We found transcriptional changes in embryos at both stages, with 79 upregulated and 48 downregulated genes in GF×CR 4-cell embryos and 223 upregulated and 179 downregulated genes in CR×GF 4-cell embryos compared to CR×CR 4-cell embryos (Figure 5). Additionally, we found 19 upregulated and 158 downregulated genes in GF×CR morulae compared to CR×CR morulae (Figure S4F). Of these genes, notably significant was *Erdr1*, which was also seen as a commonly dysregulated gene in adult somatic tissues (Figure 4). The function of *Erdr1* is thought to be related to regulation of cell death, proliferation, and migration; thus, extreme changes in *Erdr1* expression could lead to significant alterations in embryonic development.^49–51^ Overall, these data suggest that the presence or absence of microbiota leads to alterations in gametes, which correspond to downstream gene expression changes in early embryos.

### The lack of adaptive immune cells causes a transgenerational non-genetically inherited sebum secretion defect

**Figure 6.**
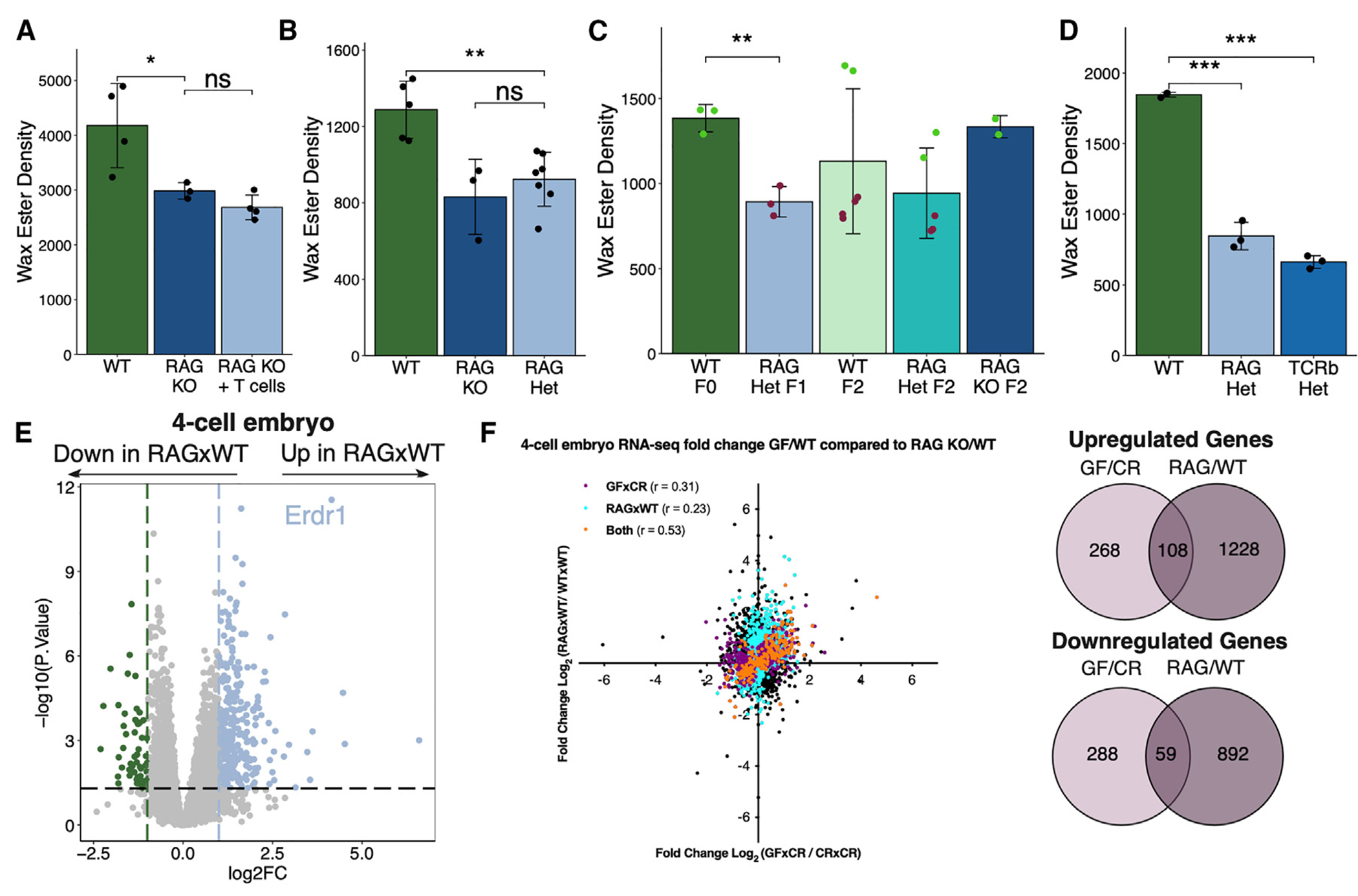
T cell-deficient mice display a sebum secretion defect that is non-genetically transmitted to progeny transgenerationally (A–D) TLC quantification of hair wax esters from (A) WT or *Rag2*^−/−^ mice with or without reconstitution by T cells (*n* = 3 or 4 mice/group). (B) WT, *Rag2*^−/−^, and F_1_ *Rag2*^+/−^ mice (*n* = 3–7 mice/group). (C) WT, F_1_ *Rag2*^+/−^, and F_2_ WT, *Rag2*^+/−^, and *Rag2*^−/−^ mice (*n* = 2–6 mice/group). Point colors represent physiologic (green) or defective (red) levels of sebum secretion. (D) F_1_ WT, *Rag2*^+/−^, and *TCRβ*^+/−^ mice (*n* = 3 mice/group). (E) Gene expression by RNA-seq of WT and RAG×WT 4-cell embryos (*n* = 21 or 25 embryos/group, collected over three biological replicates of IVF). (F) Pearson correlation analysis of GF vs. *Rag2*^−/−^ embryo gene expression on genes filtered for *p* value <0.05 (purple: significant only in GF-derived embryos; blue: significant only in *Rag2*^−/−^-derived embryos; orange: significant in both GF- and *Rag2*^−/−^-derived embryos), with DEGs shared between GF and *Rag2*^−/−^ embryos quantified. Sequencing experiments were performed once. All other experiments were performed 2 or 3 times. ns, not significant, *p < 0.05, **p < 0.01, and ***p < 0.001 by Student’s t test. Data are shown as mean ± SD. See also Figures S4 and S5.

Similar to GF mice, we have previously reported that homeostatic sebum secretion is reduced in *Rag2*^−/−^ (which lack T and B cells) and *TCRβ*^−/−^ (which lack αβ T cells) mice.^24^ To test whether this defect was acutely restorable, we adoptively transferred T cells into *Rag2*^−/−^ mice. Similar to colonization of GF mice, homeostatic sebum secretion was not restored (Figure 6A). To determine if the sebum secretion defect was transmissible to progeny, we crossed *Rag2*^−/−^ males or females to WT females or males to create F_1_ *Rag2*^+/−^ heterozygous mice, which have a normal T cell compartment.^52^ Similar to GF mice crossed to CR mice, we found that sebum secretion was defective in F_1_ *Rag2*^+/−^ mice (33 of 36 mice across 4 experiments) (Figure 6B, 100% of mice in the *Rag2*^+/−^ group from a male *Rag2*^−/−^ parent inherited the defective phenotype; Figure S5A, 100% of male and female mice in the *Rag2*^+/−^ group from a female *Rag2*^−/−^ parent inherited the defective phenotype). We next crossed the F_1_ *Rag2*^+/−^ heterozygous mice against each other to generate progeny of all 3 genotypes (*Rag2*^−/−^, *Rag2*^+/−^, and *Rag2*^+/+^). There was a mix of F_2_ progeny with either normal or defective sebum secretion, regardless of genotype; a fraction of genotypically WT mice showed defective sebum secretion, while *Rag2*^−/−^ mice showed normal sebum secretion, indicating that the phenotype of sebum secretion is not correlated with genotypes but rather with parental immune status (Figure 6C, 67% of the WT group, 60% of the *Rag2*^+/−^ group, and 0% of the *Rag2*^−/−^ group inherited the defective phenotype in the F_2_ generation; Figure S5B demonstrates confirmatory experiments, 67% [females] or 40% [males] of the WT group, 60% [females] or 75% [males] of the *Rag2*^+/−^ group, and 100% [females] or 80% [males] of the *Rag2*^−/−^ group inherited the defective phenotype in the F_2_ generation). Similar to *Rag2*^−/−^ mice, the F_1_ progeny of *TCRβ*^−/−^ mice crossed to WT mice also showed defective sebum secretion (Figure 6D, 100% of mice in the *TCRβ*^+/−^ group inherited the defective phenotype), suggesting that the absence of T cells in the F_0_ generation was responsible for the sebum secretion defect present in subsequent generations of progeny of *Rag2*^−/−^ crossed to WT mice, despite normal T cell development in these mice.

To determine whether *Rag2*^−/−^ embryos follow a similar concordance in gene expression to phenotypic change, we performed single-embryo RNA-seq on embryos generated by sperm of *Rag2*^−/−^ mice and eggs of WT mice (RAG×WT). We observed many DEGs in RAG×WT 4-cell- and morula-stage embryos, with 167 significantly changed genes shared between GF×WT and RAG×WT 4-cell embryos (Figures 6E and S4G). However, there were both commonly shared and distinct DEGs identified between GF×WT and RAG×WT 4-cell embryos, suggesting that there may be both interdependent and independent contributions of the microbiota and the adaptive immune system in controlling paternal non-genetic inheritance patterns (Figure 6F). Overall, these data suggest that similarly to microbial-dependent transgenerational epigenetic inheritance as previously shown, there also exists an immune-dependent mechanism for the transmission of the sebum secretion phenotype to successive generations.

## DISCUSSION

The results presented here describe a microbial- and immune-dependent form of transgenerational epigenetic inheritance with the ability to influence the phenotypic diversity of future generations. Our data provide evidence that the commensal microbiota is not only important for acute changes in organ function but can also have a persistent effect on future generations. We also describe a unique and impactful role of the immune system in influencing gametes to alter the control of gene expression and phenotypes of succeeding generations of progeny.

There are many examples illustrating the importance of host-microbe interactions in regulating functional biological processes.^23,36,37^ As such, there are innumerable defects present in the tissues of GF mice ranging from barrier sites to internal organ systems.^23,46,53^ Reversal of these GF defects with bacterial colonization is a common experimental tool in microbiome research, though some GF phenotypes are not acutely reversible with colonization, for which there is no explanation. From the work we describe, we propose that the dichotomy in the reversal of GF phenotypes is due to an important distinction in acute phenotypic changes vs. persistent non-genetically inherited phenotypes. As we have demonstrated striking evidence that a dysregulated GF transcriptome in multiple tissues is passed across generations, it will be important to match the transcriptional changes to phenotypic function of these tissues to determine their effects. For example, the gut immune function of GF×CR F_1_ mice is likely to be perturbed given the reduction in transcriptional programs that control innate bacterial response in the small intestine of CONV GF-derived F_1_ mice.

**Figure 7.**
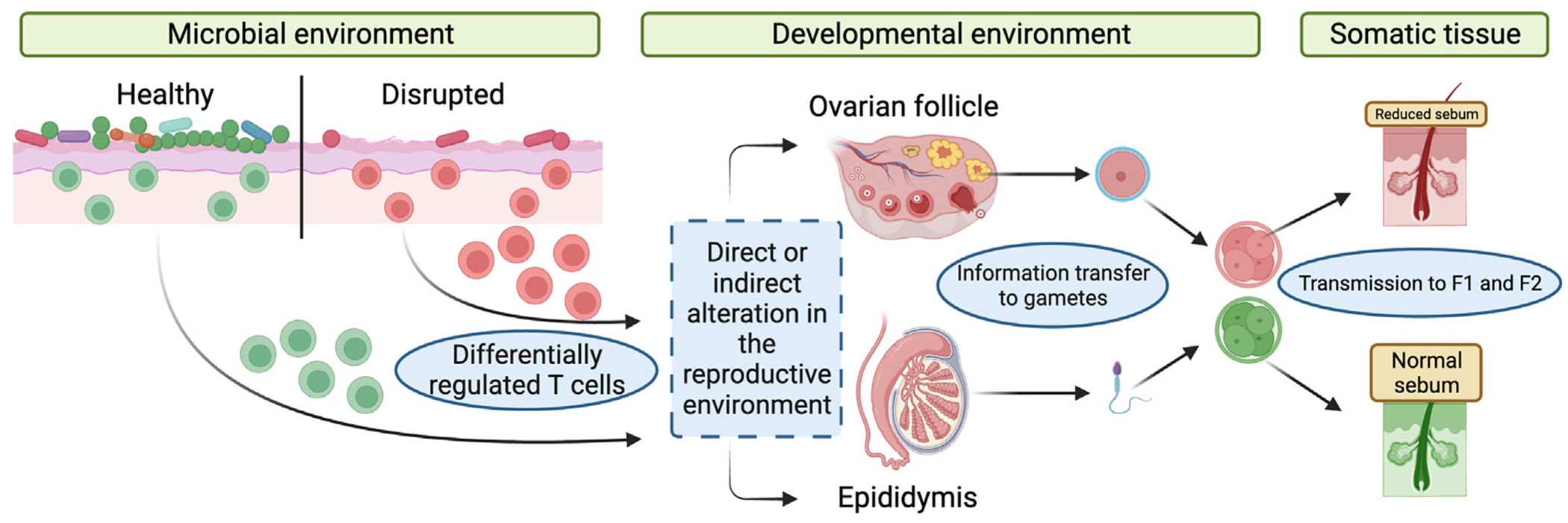
Microbial and immune alterations lead to heritable and persistent phenotypic diversity in progeny Model for microbial- and immune-mediated transgenerational epigenetic inheritance. Environmental perturbations lead to alterations in the microbiota and barrier immune cell population. These shifts affect the epigenetic information in gametes, which then lead to downstream differential embryonic gene expression and somatic phenotypes in adult mice.

While we hypothesized that microbes use T cells as messengers to communicate with reproductive tissues, we did not observe a perfect correlation between the gene expression changes in progeny resulting from the lack of microbiota and T cells, suggesting that there are independent effects of both systems in controlling transgenerational phenotypes. Yet, based on many similarities in the inheritance pattern and related inherited modulation of embryonic gene expression, we propose a model (Figure 7) whereby the microbial environment, at least in part, is detected by T cells, which then transfer this information, directly or indirectly, to the reproductive tissues, thereby altering epigenetic information in the gametes and transmitting phenotypic diversity to future generations.

It has been shown that the microbial composition of barrier sites is altered in response to the environment.^19,54^ Thus, environmental perturbations could be sensed by changes in commensal microbial composition, which are then provided as information to offspring to adapt more successfully to the environment. Moreover, previous reports in mice have described how diet alterations and stress program non-genetically inherited phenotypes in subsequent generations of progeny.^11,12,55^ The microbiome and the immune system have been independently linked to both changes in diet and stress.^16,22,56^ Thus, it is plausible that modifications in diet or the introduction of persistent stress and the resulting microbial and immune alterations are responsible for altering epigenetic information in the gametes and intergenerational information transfer. Teleologically, we believe our observations suggest that microbial presence can provide environmental context to offspring to allow for optimal use of energy and metabolism. As an example, we show that F_0_ and F_1_ GF mice have reduced sebum secretion and that F_1_ livers show altered metabolic lipid processing. It is enticing to speculate that because of the absence of microbes in GF mice, the host is shunting metabolic effort normally reserved for sebum secretion and barrier function to the liver to save energy.

Despite numerous examples in model organisms ranging from plants to *C. elegans*,^17^ the existence of transgenerational non-genetic inheritance in mammals has been controversial. This controversy is a result of weakly penetrant and expressive phenotypes that have been demonstrated to be transmitted by transgenerational epigenetic inheritance and undefined molecular mechanisms underlying the phenomenon. Only recently has evidence of this pattern of inheritance contributing to mammalian phenotypes been uncovered, although these studies have not completely resolved the controversy.^9,13–15,18,57–62^ For example, it has recently been discovered that changes in small non-coding RNA (ncRNA) in sperm can lead to alterations in embryonic gene regulation and phenotypes of future generations.^11,12,63–65^ In particular, the tRNA fragments (tRFs) Gly-GCC and Val-CAC and a subset of microRNAs (miRNAs) have been shown to be delivered to sperm by fusion with extracellular vesicles, called epididymosomes.^11^ Further, tRF-Gly-GCC and epididymally acquired miRNAs have been demonstrated to regulate embryonic gene expression post-fertilization, as well as to program offspring phenotypes.^12,66^ This phenomenon is an appealing method of transmission potentially related to our findings, as it would allow for immune cells influenced by microbial alterations to influence gametic RNA content based on the environment, promoting differential genotypes and phenotypes in offspring. However, multiple mechanisms of inheritance could contribute to these transgenerational findings. Additional possibilities include other modes of non-genetic influence including the idea that immune cells could alter chromatin architecture or DNA methylation characteristics in gametes, leading to persistent downstream effects in progeny.^67,68^ As such, future investigation will focus on uncovering the mechanism of epigenetic information transfer from the microbiome and immune cells to gametes, embryos, and adult tissue of progeny. To accomplish the goal of determining a mechanism of transgenerational epigenetic inheritance in mammals, we believe it is important that in this work, we describe a robust readout of the non-genetic inheritance patterns using sebum secretion, providing a sensitive model for groundbreaking studies to understand the molecular mechanisms underlying the transfer of epigenetic information between generations and throughout development.

From our studies, we observe that the gene *Erdr1* is strikingly upregulated in both GF-derived early embryos and adult somatic tissues. Interestingly we also find that *Erdr1* is regulated analogously in early embryos derived from eggs of WT mice fertilized by sperm of *Rag2*^−/−^ mice. While the significance of these observations is currently unknown, *Erdr1* poses as an intriguing target for future studies of microbial-dependent transgenerational phenotypes potentially acting as a common thread across generations.

As a result of the discovery of this microbial-immune-transgenerational phenotypic inheritance, we could contextualize events from the past, attempt to explain the state of human health in the present, and learn how our current decisions could affect the future. In the modern era, one of the most significant changes in human health is the explosive onset of atopic and autoimmune disease. The “hygiene hypothesis” is a popular idea to explain how the prevalence of atopy and autoimmunity have risen whereby human society has become more hygienic and less barraged by pathogens to train the immune system, leading to immune overactivation in the form of allergy and autoimmunity.^69,70^ We might consider that the effects of sanitation from industrialization have been passed down over multiple generations and are increasingly materializing in the modern day in the form of immune dysregulation. As a form of positive adaptation, this phenomenon may be a way for mammals to introduce phenotypic diversity into their offspring due to environmental change without the long-term necessity of genetic-mutation-based natural selection. In this way, animals would have an increased chance at quickly and persistently adapting to new environmental threats looming on the horizon.

### Limitations of the study

Our study describes a phenomenon whereby the murine microbial and immune environment can have a significant impact on the gene expression and phenotypic landscape of multiple organ systems in subsequent generations. Although our data suggest that the phenotypes are not transmitted genetically, we have not identified the epigenetic mechanism by which the information transfer occurs from the parental generation to the F_1_ progeny and beyond. Previous studies have suggested mechanisms of transgenerational inheritance via epigenetic means, including small ncRNAs, DNA methylation, or chromatin architecture.^11,67,68^ Follow-up studies will aim to elucidate the epigenetic mechanisms that control microbe and immune-mediated transgenerational epigenetic inheritance. Further, while analyzing the RNA-seq data of multiple tissues comparing CR×CR and GF×CR, we acknowledge that we performed gene detection on both multiple-comparison-corrected and uncorrected p values as an exploratory technique, as we are comparing tissue from unmanipulated mice reared in the same facility. We report these findings in the main and supplemental figures, although the patterns of gene transcription changes remain the same in both analyses. Lastly, our work mainly focuses on skin microbiota and skin phenotypes, as SG activity was found to be a robust readout to track the transgenerationally inherited phenotype. However, we also found gene expression alterations in other organ systems such as gut and liver, but currently, we do not know what phenotypes these transcriptional changes lead to. Thus, future studies will involve phenotypic readouts in multiple organ systems to further expand the evidence of microbial- and immune-mediated transgenerational epigenetic inheritance.

## STAR★METHODS

### RESOURCE AVAILABILITY

#### Lead contact

Further information and requests for resources and reagents should be directed to and will be fulfilled by the lead contact, Taku Kambayashi (taku.kambayashi@pennmedicine.upenn.edu).

#### Materials availability

This study did not generate unique reagents.

#### Data and code availability

- All RNA sequencing data generated from this study have been deposited at GEO and are publicly available as of the date of publication from accession number GSE240797. Microscopy data reported in this paper will be shared by the lead contact upon request.
- Code used to analyze RNA sequencing data is derived from the pipeline reported by Berry et al.^80^ and freely available at https://diytranscriptomics.com/.
- Any additional information required to reanalyze the data reported in this work paper is available from the lead contact upon request.

### EXPERIMENTAL MODEL AND STUDY PARTICIPANT DETAILS

All specific pathogen-free (conventionally raised: CR) mice used in these studies were derived from C57BL/6 mice purchased from Charles River Laboratories (strain number 556) unless otherwise specified. Germ-free (GF) mice were obtained from the University of Pennsylvania Gnotobiotic Core, which houses C57BL/6 and Swiss-Webster colonies in sterile isolators. Additional GF mice were obtained from the Gnotobiotic Core at University of North Carolina at Chapel Hill for comparing colony phenotypes. CR and GF breeding pairs were established in a conventional mouse facility at the University of Pennsylvania. *Rag2*^−/−^ mice (Jackson Laboratories strain number 008449), *TCRβ*^−/−^ mice (Jackson Laboratories strain number 002116), and *Scd1*^−/−^ mice (Jackson Laboratories strain number 006201) were obtained from Jackson Laboratories and bred within our mouse facility. *Rag2*^+/−^, *TCRβ*^+/−^, and *Scd1*^+/−^ mice were derived in our animal facility by breeding the knockout strains to C57BL/6 wild-type mice (Charles River strain number 556). Unless otherwise specified, all mice used in these studies were 8 weeks old at the time of use. A combination of both male and female mice was used in the studies to ensure conclusions could be generalized to both sexes. All mice were housed in either specific pathogen-free or germ-free conditions and were handled under strict compliance with the University of Pennsylvania Institutional Animal Care and Use Committee regulations.

### METHOD DETAILS

#### Lipid extraction and thin-layer chromatography

To isolate sebum lipids from mouse fur, a standardized 3 cm × 3 cm area of fur was shaved from the back. Fur was submerged in 2 mL of 2:1 (v/v) chloroform:methanol (Sigma-Aldrich 288306 and Sigma-Aldrich 322415) followed by sonication in a water bath for 6 min to dislodge lipids, and syringe filtration to remove fur from solution. Fur was then submerged in 2 mL of acetone and sonication and filtration steps were repeated. The organic solution containing fur lipids was evaporated using nitrogen gas until completely dry and dissolved in 250 μL of 4:1 chloroform:methanol (v/v). 5 μL of lipid solution was then loaded onto a thin-layer chromatography plate (Sigma-Aldrich, 100390) and placed sequentially in (1) a shallow solution of 80:20:1 hexane (Sigma-Aldrich 296090):diisopropyl ether (Sigma-Aldrich 673803):acetic acid to migrate to a plate height of 50%, (2) a shallow solution of 1:1 hexane:benzene (Sigma-Aldrich 401765) to migrate to a plate height of 80%, and (3) a shallow solution of hexane to migrate to a plate height of 90%, with 15 min of drying time between each migration step. Plates were then uniformly coated with 10% copper (II) sulfate (Sigma-Aldrich 451657)/8% phosphoric acid (Sigma-Aldrich 345245) solution, allowed to dry, and baked at 120°C for 20 min to visualize lipid species. Adobe Photoshop was used to quantify the integrated density of the lipid bands. As a standard for lipid species identification, the TLC non-polar lipid mixture A (Cayman Chemical 29377) was used.

#### Skin RNA extraction and cDNA synthesis for qPCR

On the day of tissue harvest, fur was shaved, back skin was removed, minced, snap frozen and stored at −80°C until further processing. To isolate RNA from skin, frozen tissue was transferred to TissueTube TT05M XT tissue bags (Covaris 520140) and pulverized using a Covaris automated dry pulverizer (Covaris CP02) by submerging the tissue bag in liquid nitrogen for 10 s and immediately transferring for pulverization. Pulverized tissue was then transferred to 1 mL of TRIzol (ThermoFisher 15596026) and RNA extracted according to the TRIzol manufacturer’s protocols. Glycogen (ThermoFisher AM9510) was used as a carrier during extraction. A Nanodrop 1000 was used to quantify isolated RNA. Following RNA extraction, cDNA was synthesized using Superscript Vilo (ThermoFisher 11754050) according to the manufacturer’s instructions. Quantitative polymerase chain reaction (qPCR) was then performed using the Taqman Fast Advanced Master Mix (ThermoFisher 4444557) according to the manufacturer’s instructions, with the following primer from Taqman: *Tslp* (Mm01157588_m1). qPCR reactions were performed using a ViiA7 Real-Time PCR instrument (ThermoFisher).

#### Flow cytometry

To quantify T cells in ear skin, dermal sheets were separated, and finely minced in RPMI 1640 media (ThermoFisher 11875093) complemented with 10% fetal bovine serum (R&D Systems S11150) (cRPMI) containing 100 μg/mL of Liberase TL (Roche 5401020001) and 50 μg/mL of DNase I (Sigma-Aldrich DN25). Minced tissue was incubated with shaking at 37°C for 1 h and then strained through a 70 μm filter into a new tube containing 1 mL cRPMI. Cells were stained with cell surface stains and live-dead stain at 4°C for 15 min in PBS. Flow cytometry was then performed using an LSR II or LSR Fortessa instrument (BD Biosciences). Compensation was performed using compensation beads (BD Biosciences 552845). Flow cytometry data was analyzed using FlowJo software (BD Biosciences). Staining antibodies used included CD45.2 (mouse, PE fluorochrome, clone 104, BD Biosciences 560695, 1:200 dilution), TCRβ (mouse, PE-Cy7 fluorochrome, clone H57-597, BioLegend 109222, 1:200 dilution), CD4 (mouse, FITC fluorochrome, clone RM4-5, BioLegend 100510, 1:200 dilution), CD8a (mouse, PerCP-Cy5.5 fluorochrome, clone 53–6.7, BioLegend 100734, 1:200 dilution) and Live/Dead Near-IR (ThermoFisher L10119, 1:1000 dilution). CountBright beads were used for counting cells and normalization (ThermoFisher C36950).

#### Laser capture microdissection, RNA extraction, and sequencing

Mouse back skin from CR, GF, CR×CR F_1_, and GF×GF F_1_ was collected and fixed overnight in 4% paraformaldehyde (Fisher AAJ19943K2) at 4°C followed by paraffin embedding. Laser capture microdissection (LCM) was performed using the LMD 7000 system (Leica Microsystems). FFPE mouse skin was processed and cut onto a polyethylene naphthalate (PEN) slide designed for LCM processing (Leica 11505158). At least 1,000 SGs or 1,000,000 μm^2^ of tissue was isolated to obtain enough material for RNA extraction. SG RNA was extracted from post-LCM tissue using a Qiagen All Prep DNA/RNA FFPE Kit (Qiagen 80234). RNA concentration was measured by Qubit fluorometric quantification (ThermoFisher Qubit 2.0 Fluorometer) and RNA quality measured via BioAnalyzer (Agilent 2100 Bioanalyzer Instrument). cDNA libraries were prepared using Illumina Stranded Total RNA Prep with Ribo-Zero Plus Kit (Illumina 20040529) with IDT for Illumina RNA UD Indexes, Set A (Illumina 20040553). Libraries were assessed for cDNA quantity and library quality using Qubit and BioAnalyzer. As necessary, an extra bead wash step was performed to remove excess primer dimers in the library and purify samples further. Samples were then pooled and sequenced on a Nextseq 550 using a NextSeq 500/550 High Output Kit v2.5 (150 Cycles) (Illumina 20024907).

#### Somatic tissue RNA-seq analysis

Transcriptomic analysis of sebaceous glands, skin, small intestine, and liver was performed in the R statistical computing environment version 4.2 and RStudio version 2022.02.1 using a pipeline adopted from an open-source toolkit for RNA sequencing analysis.^80^ For pseudoalignment of reads to a reference genome, Kallisto was used in combination with the Ensembl species-specific database for gene annotation.^71^ A filtration cutoff was used of 1 count per million in the number of samples equal to the *n* of the smallest group. Data was normalized using the Trimmed Mean of M-values (TMM) method from the EdgeR package.^72^ Post-filtered, post-normalized data was then variance stabilized using the VOOM function from the Limma package.^73^ Limma was then used for differential gene expression (DGE) testing with multiple testing correction via the Benjamini-Hochberg method.^81^ For F_1_ SG samples, DGEs were defined as genes with BH-adjusted p value <0.05. For other somatic F_1_ and F_2_ samples a less stringent cut-off was used to define DGEs as genes with p value <0.05, as we were testing for broad similarities between cross-generational transcriptomic profiles. Gene ontology (GO) analysis was performed using the gprofiler2 R package^74^ with terms identified from the GO knowledgebase with FDR adj-p-value <0.05 and gene set enrichment analysis was performed using the msigdbr and clusterprofiler R packages.^75,76,82^

#### Bacterial colonization and culture

To colonize germ-free mice with a conventional microbiota, 8-week-old germ-free mice were transferred to a conventional specific pathogen-free mouse facility and were exposed to bedding and cage material from three other mature mouse cages three times in the first week of transfer. The conventionalized germ-free mice had weekly cage changes, thus allowing for further microbial exposure. These mice were housed in this manner for eight weeks prior to takedown at which point mice were swabbed for bacterial culture and confirmation of adequate colonization. Swabs (Puritan 25–1506) were dipped in PBS before deeply swabbing pre-shaved mouse back fur 10–15 times. Swabs were stored in PBS at RT for 30 min and then serially diluted for plating on blood agar (Thermo Scientific R01200). Colony forming units (CFUs) quantified by counting number of colonies on blood agar at a dilution with colony number between 10 and 100 and calculated based on dilution and volume used for plating.

#### Microbial 16S rRNA gene sequencing

##### Skin microbiome sample collection and DNA extraction

Skin microbiome samples were collected using individually wrapped sterile swabs (Puritan 25–1506) dipped in sterile PBS followed by deeply swabbing the back of mice 10–15 times. Swabs were then stored in individually wrapped, sterile Eppendorf Safe-Lock tubes (Eppendorf 022600044) at −80°C until DNA extraction. Genomic DNA was extracted from skin swabs as described in Meisel et al., 2016.^83^ Briefly, each swab was incubated at 37°C for 1 h continuously shaking in 300 μL yeast cell lysis solution (Biosearch Technologies MasterPure Yeast DNA Purification kit #MYP80200) in addition to 10,000 units of ReadyLyse Lysozyme solution (Biosearch Technologies #R1810M). Samples were processed using bead beating for 10 min at maximum speed on a vortex with 0.5 mm glass beads (Qiagen #13116-50). After a 30-min incubation at 65°C with shaking, protein precipitation reagent was added, and samples were spun at maximum speed. The supernatant was removed, mixed with isopropanol, and applied to a PureLink Genomic DNA Mini Kit column (Invitrogen #K182002). The columns were washed with Buffer 1 and 2 before eluting the DNA using 50 μL MilliQ sterile water. Swab control samples were prepared and sequenced exactly as the experimental samples.

##### Gut microbiome sample collection and DNA extraction

Gut microbiome samples were collected by isolating 1–2 individual fecal pellets from mice of interest. These pellets were stored in individually wrapped, sterile Eppendorf Safe-Lock tubes (Eppendorf 022600044) at −80°C until DNA extraction. Genomic DNA was extracted from fecal samples using a Qiagen DNeasy PowerSoil Pro kit as described by the manufacturer’s instructions (Qiagen 47014).

##### Fecal and skin swab samples 16S rRNA gene sequencing

The 35 samples were prepared using the automated amplification and sequencing system by Seq Center (Pittsburgh, PA). The amplification process was performed from DNA using Zymo Research’s Quick-16S kit with phased primers targeting the V3/V4 regions of the 16S rRNA gene. The specific sequences for the forward primers used were CCTACGGGDGGCWGCAG and CCTAYGGGG YGCWGCAG; and GACTACNVGGGTMTCTAATCC for the reverse primer. Following clean up and normalization, samples were sequenced on a P1 600cyc NextSeq2000 Flowcell to generate 2x301bp paired end (PE) reads. Quality control and adapter trimming was performed with bcl-convert1 (v4.2.4).

##### 16S rRNA amplicon sequencing analysis

Sequences were processed using QIIME 2 pipeline.^77^ A total of 1,919,298 and 996,260 demultiplexed 300 base PE reads from skin swabs and fecal samples respectively, were imported using Casava 1.8 format and denoised using DADA2 to obtain an amplicon sequence variant (ASV) table.^78,84^ Singletons (ASV present <2 times) and ASVs that are present in less than 10% of the samples were discarded. Greengenes reference sequences (clustered at 99% similarity) were used to train a naive Bayes taxonomy classifier to further annotate ASVs taxonomically.^79^ ASVs were then collapsed based on genus or lowest-level (i.e., family, order, class, phylum) taxonomy possible. An even sampling depth of 4323 and 1907 sequences per sample was used for assessing alpha- and beta-diversity measures in the skin swabs and fecal samples, respectively. Evenness diversity Index and Faith’s phylogenetic diversity (PD) was used to measure alpha diversity.

#### TSLP-AAV injections

Two adeno-associated virus vectors used in these studies were generated by the Penn Vector Core, including Control-AAV (AAV8.TBG.PI.eGFP.WPRE.bGH) and TSLP-AAV (AAV8.TBG.PI.mTSLP.IRES.eGFP.WPRE.bGH). Doses were previously optimized and mice were intravenously injected with 5×10^10^ genome copies of both Control- and TSLP-AAV for 14 days with serum TSLP levels confirmed using a murine specific ELISA (R&D Systems MTLP00).^24^

#### Histology

Skin tissue was isolated from mouse back and fixed at 4°C overnight in 4% paraformaldehyde (Fisher AAJ19943K2) prior to paraffin embedding. Processing and staining (H&E) was performed by the University of Pennsylvania’s Skin Biology and Disease Resource Center. For H&E skin sections, full section stitching at 40× magnification was performed to image one full section of skin per biological replicate. Within these sections, a total of 27–53 SGs were measured. For measurement of SG area, ImageJ (NIH) was used to draw circumscribing ellipses around SG edges. Samples were imaged using a Keyence VHX-6000 digital microscope system and were prepared for publication using ImageJ and Photoshop.

#### Adoptive transfers

T cell adoptive transfers were performed by isolating CR or GFT cells for intravenous injection into *Rag2*^−/−^ mice. Splenic T cells were isolated for transfer using a T cell negative selection kit (STEMCELL Technologies 19851) according to the manufacturer’s instructions. Intravenous injection of 5×10^6^ isolated T cells was then performed and six weeks later sebum secretion was measured.

#### Bulk RNA extraction and sequencing

*Skin*: On the day of tissue harvest, fur was shaved, back skin was removed, minced, snap frozen and stored at −80°C until further processing. RNA was extracted from skin using the same method as described above in preparation for qPCR. *Small intestine*: On the day of tissue harvest, 1 cm of distal ileum was snap frozen and stored at −80°C until further processing. Tissue was homogenized in tubes with metal beads using TRIzol extraction as detailed previously. *Liver*: On the day of tissue harvest, liver tissue was snap frozen and stored at −80°C until further processing. RNA was extracted from tissue using TRIzol extraction as detailed previously.

Quality control of RNA was performed using a Qubit fluorometer (ThermoFisher Qubit 2.0 Fluorometer) for quantification and Bioanalyzer (Agilent 2100 Bioanalyzer Instrument) or TapeStation (Agilent 4200) for RNA quality. *Skin*: cDNA libraries were prepared using the Illumina Stranded Total RNA Prep with Ribo-Zero Plus Kit (Illumina 20040529) with IDT for Illumina RNA UD Indexes, Set A (Illumina 20040553) and sequenced on a NovaSeq 6000 using a NovaSeq 6000 SP Reagent Kit v1.5 (100 cycles) (Illumina 20028401). *Small intestine*: Libraries were prepared using the Illumina Stranded mRNA Prep, Ligation kit according to the manufacturer’s instructions. Unique Illumina TruSeq dual indices were used for sample identification. Library pool was sequenced on an Illumina NextSeq 550 instrument using 75 cycles, single-end. *Liver*: mRNA-sequencing libraries were generated using Illumina stranded mRNA kit as per manufacturer’s instructions. Paired-end sequencing was performed using Illumina NextSeq 1000. Data were mapped using RSEM and normalized using transcripts per million (tpm).

#### Egg collection, *in vitro* fertilization, and embryo culture and transfer

Eggs were retrieved from the ampullae of 4- to 6-week-old female mice following superovulation as previously described.^85^ For egg collection for small RNA sequencing, cumulus oocyte complexes (COCs) were incubated in hyaluronidase (1 mg/mL) to dissociate cumulus cells from eggs. Eggs were washed through six droplets of KSOM to remove any residual cumulus cells and collected in 1×TCL buffer (supplemented with 1% β-mercaptoethanol). For *in vitro* fertilization (IVF), spermatozoa were collected and capacitated as previously described.^85^ Spermatozoa (2 × 10^5^) were added to the IVF droplet and co-incubated with eggs for 3 h at 37°C under an atmosphere of 5% O_2_, 6% CO_2_. Presumptive zygotes were washed in KSOM and cultured until 2-cell (24 h), 4-cell (46 h) and morula (72 h) stage.

Transfer of IVF generated embryos was performed at the Children’s Hospital of Philadelphia Transgenic Core. Embryos cultured to the 2-cell stage of development were transferred to the oviduct of pseudopregnant recipient females to produce live pups.

#### Embryo mRNA-sequencing

Single embryos, sired by control, germ-free or *Rag2*^−/−^ sperm or eggs from control or GF mice were collected for single-embryo/egg RNA-sequencing. Embryos were transferred to a 96-well plate and fresh 1 × TCL buffer with 1% β-mercaptoethanol was added. RNA was size selected using RNA-Clean XP beads (Beckman Coulter A63987) and full-length polyadenylated RNA was reverse transcribed using Superscript II. Resulting cDNA was amplified using 10 cycles and the amplified product was used to construct a pool of uniquely indexed samples using the Nextera XT kit (Illumina FC-131-1096). Finally, pooled libraries were sequenced by Illumina NextSeq1000 (paired end).

### QUANTIFICATION AND STATISTICAL ANALYSIS

All data reported are represented as mean ± standard deviation. All measurements were made using distinct biological replicates and experiments characterizing individual sebaceous glands included several technical replicates per biological replicate. Prior experience in the lab on the number of mice needed to reach statistical significance in addition to mouse availability was used to determine sample sizes. All data being used in statistical comparisons were verified as normal using the Shapiro-Wilk measure of normality, and thus statistical significance was determined by a two-sided Student’s *t* test. Correlation analyses in Figure 6 were performed using a Pearson correlation. All statistical analyses were performed using the R statistical computing environment version 4.2 and RStudio version 2022.02.1. For LCM-isolated sebaceous gland transcriptional analyses, *p* values were adjusted using the Bonferroni-Hochberg method and differential expression was determined as a gene with BH-adj *p* value <0.05 and log_2_-fold change >1 or < −1. For epidermal transcriptional analysis^23^ of control, germ-free, and conventionalized mice, the gene list of DEGs comparing CONV vs. GF was used to remove any shared DEGs in the CR vs. GF DEG list, leaving a gene list of “persistent” genes in GF mice. Here, DEGs a cutoff of FDR-*p*-adjusted <0.1. For more exploratory analyses of global gene and pathway changes across generations in skin, small intestine, and liver, a less stringent cutoff was used of a non-adjusted *p* value <0.05 and log_2_-fold change >1 or < −1. Analyses using traditional adjusted *p* values are included in Figure S4. For embryonic gene expression analyses, we also used a less stringent cutoff of a non-adjusted *p* value <0.05 and log_2_-fold change >1 or < −1. All GO analyses were performed using an FDR-corrected *p*-value <0.05. All GSEA analyses were performed using a BH-corrected p value <0.05. Correlation plot in Figure 6F did not use a log_2_-fold change cut-off, to best show correlation of expression across all significant genes.

## Supplementary Material

1

## Figures and Tables

**Table:** KEY RESOURCES TABLE

REAGENT or RESOURCE	SOURCE	IDENTIFIER
Antibodies		
PE Mouse Anti-Mouse CD45.2	BD Biosciences	Cat#560695; RRID:AB_1727493
PE/Cyanine7 anti-mouse TCR β chain Antibody	BioLegend	Cat#109222; RRID:AB_893627
FITC anti-mouse CD4 Antibody	BioLegend	Cat#100510; RRID:AB_312713
PerCP/Cyanine5.5 anti-mouse CD8a Antibody	BioLegend	Cat#100734; RRID:AB_2075239
Chemicals, peptides, and recombinant proteins		
Chloroform	Sigma-Aldrich	Cat#288306
Methanol	Sigma-Aldrich	Cat#322415
Hexane	Sigma-Aldrich	Cat#296090
Diisopropyl ether	Sigma-Aldrich	Cat#673803
Acetic acid	Sigma-Aldrich	Cat#695092
Benzene	Sigma-Aldrich	Cat#401675
Copper (II) sulfate	Sigma-Aldrich	Cat#451657
Phosphoric acid	Sigma-Aldrich	Cat#345245
TLC non-polar lipid mixture A	Cayman Chemical	Cat#29377
TRIzol reagent	ThermoFisher	Cat#15596026
Glycogen	ThermoFisher	Cat#AM9510
Taqman Fast Advanced Master Mix	ThermoFisher	Cat#4444557
RPMI 1640 media	ThermoFisher	Cat#11875093
Fetal bovine serum	R&D Systems	Cat#S11150
Liberase TL	Roche	Cat#5401020001
Deoxyribonuclease I from bovine pancreas	Sigma-Aldrich	Cat#DN25
CompBeads Anti-Rat and Anti-Hamster Ig κ/Negative Control Compensation Particles	BD Biosciences	Cat#552845
CountBright Absolute Counting Beads	ThermoFisher	Cat#C36950
4% Paraformaldehyde	Fisher Scientific	Cat#AAJ19943K2
ReadyLyse Lysozyme solution	Biosearch Technologies	Cat#R1810M
RNAClean XP Beads	Beckman Coulter	Cat#A63987
Critical commercial assays		
SuperScript VILO cDNA Synthesis Kit	ThermoFisher	Cat#11754050
LIVE/DEAD Fixable Near-IR Dead Cell Stain Kit	ThermoFisher	Cat#L10119
AllPrep DNA/RNA FFPE Kit	Qiagen	Cat#80234
Illumina Stranded Total RNA Prep, Ligation with Ribo-Zero Plus	Illumina	Cat#20040529
IDT for Illumina RNA UD Indexes Set A, Ligation	Illumina	Cat#20040553
NextSeq 500/550 High Output Kit v2.5 (150 Cycles)	Illumina	Cat#20024907
MasterPure Yeast DNA Purification Kit	Biosearch Technologies	Cat#MPY80200
PureLink Genomic DNA Mini Kit	Invitrogen	Cat#K182002
DNeasy PowerSoil Pro Kit	Qiagen	Cat#47014
Quick-16S^™^ NGS Library Prep Kit	Zymo Research	Cat#D6400
NextSeq 1000/2000 P1 Reagents(600cycles)	Illumina	Cat#20075294
Mouse TSLP Quantikine ELISA Kit	R&D Systems	Cat#MTLP00
EasySep Mouse T cell Isolation Kit	Stemcell Technologies	Cat#19851
NovaSeq 6000 SP Reagent Kit v1.5 (100 cycles)	Illumina	Cat#20028401
Nextera XT DNA Library Preparation Kit	Illumina	Cat#FC-131-1096
Deposited data		
Generated RNA-sequencing data	Gene Expression Omnibus	GEO: GSE240797
Experimental models: Organisms/strains		
Mouse: C57BL/6 WT	Charles River	Strain #556
Mouse: Germ-free C57BL/6 UPenn	University of Pennsylvania	GF-B6
Mouse: Germ-free Swiss-Webster	University of Pennsylvania	GF-SW
Mouse: Germ-free C57BL/6 UNC	University of North Carolina-Chapel Hill	UNC GF-B6
Mouse: C57BL/6 *Rag2*^−/−^	Jackson Laboratories	Strain #008449
Mouse: C57BL/6 *TCRβ*^−/−^	Jackson Laboratories	Strain #002116
Mouse: C57BL/6 *Scd1*^−/−^	Jackson Laboratories	Strain #006201
Oligonucleotides		
Taqman *Tslp* murine assay	ThermoFisher	Mm01157588_m1
Recombinant DNA		
Control-AAV	University of Pennsylvania Vector Core	AAV8.TBG.PI.eGFP.WPRE.bGH
TSLP-AAV	University of Pennsylvania Vector Core	AAV8.TBG.PI.mTSLP.IRES.eGFP.WPRE.bGH
Software and algorithms		
Adobe Photoshop 2023	Adobe	https://www.adobe.com/products/photoshop.html
FlowJo Software version 10.10	BD Biosciences	https://www.flowjo.com/solutions/flowjo
R statistical computing environment version 4.2	R	https://www.r-project.org/
RStudio version 2022.02.1	Posit	https://posit.co/download/rstudio-desktop/
Kallisto pseudoalignment program	Bray et al.^71^	https://pachterlab.github.io/kallisto/
R package: edgeR	Robinson et al.^72^	https://bioconductor.org/packages/release/bioc/html/edgeR.html
R package: Limma	Ritchie et al.^73^	https://bioconductor.org/packages/release/bioc/html/limma.html
R package: gprofiler2	Kolberg et al.^74^	https://cran.r-project.org/web/packages/gprofiler2/index.html
R package: msigdbr	Liberzon et al.^75^	https://cran.r-project.org/web/packages/msigdbr/index.html
R package: clusterprofiler	Wu et al.^76^	https://bioconductor.org/packages/release/bioc/html/clusterProfiler.html
QIIME 2	Boylen et al.^77^	https://qiime2.org/
DADA2	Callahan et al.^78^	https://benjjneb.github.io/dada2/
Greengenes reference database	McDonald et al.^79^	https://greengenes.secondgenome.com/
ImageJ 1.52q	NIH	N/A
Adobe Illustrator 2023	Adobe	https://www.adobe.com/products/illustrator.html
Biorender	Biorender	https://www.biorender.com/
Other		
Thin-Layer Chromatography plate	Sigma-Aldrich	Cat#100390
TissueTube TT05M XT tissue bags	Covaris	Cat#520140
cryoPREP Automated Dry Pulverizer (110V)	Covaris	Cat#CP02
ViiA7 Real-Time PCR	ThermoFisher	Cat#4453536
LSR Fortessa cell analyzer	BD Biosciences	N/A
LMD 7000 Laser Capture Microdissection system	Leica Microsystems	LMD7000
Polyethylene naphthalate LCM slides	Leica Microsystems	Cat#11505158
Qubit 2.0 Fluorometer	ThermoFisher	N/A
2100 Bioanalyzer Instrument	Agilent	N/A
NextSeq 550 System	Illumina	N/A
NextSeq 1000 System	Illumina	N/A
NextSeq 2000 System	Illumina	N/A
NovaSeq 6000 System	Illumina	N/A
6″ Sterile Standard Foam Swab w/Polystyrene Handle	Puritan	Cat#25-1506
Blood Agar (TSA with Sheep Blood) Medium	Thermo Scientific	Cat#R01200
Eppendorf Safe-Lock Tubes	Eppendorf	Cat#022600044
PowerBead Tubes, Ceramic 1.4 mm	Qiagen	Cat#13113-50
Keyence VHX-6000 digital microscope system	Keyence	VHX-6000
TapeStation 4200	Agilent	N/A

## Highlights

- Germ-free and T cell-deficient mice show defects in barrier tissue function
- Defects persist transgenerationally via non-genetic inheritance
- Immune-microbe-influenced inheritance is transmitted by the germlines of both sexes
- The microbiome and immune system impact embryonic gene expression of progeny
