# Supplementary material for: The microbiota and T cells non-genetically modulate inherited phenotypes transgenerationally
Source: Cell Rep. Author manuscript; Available in PMC 2024 May 18. (PMC11102039; doi:10.1016/j.celrep.2024.114029)
Supplement: 1 [file NIHMS1988696-supplement-1.pdf]

Cell Reports, Volume *43*

Supplemental information

The microbiota and T cells non-genetically modulate inherited phenotypes transgenerationally

Jordan C. Harris, Natalie A. Trigg, Bruktawit Goshu, Yuichi Yokoyama, Lenka Dohnalová, Ellen K. White, Adele Harman, Sofía M. Murga-Garrido, Jamie Ting-Chun Pan, Preeti Bhanap, Christoph A. Thaiss, Elizabeth A. Grice, Colin C. Conine, and Taku Kambayashi

Supplemental Items

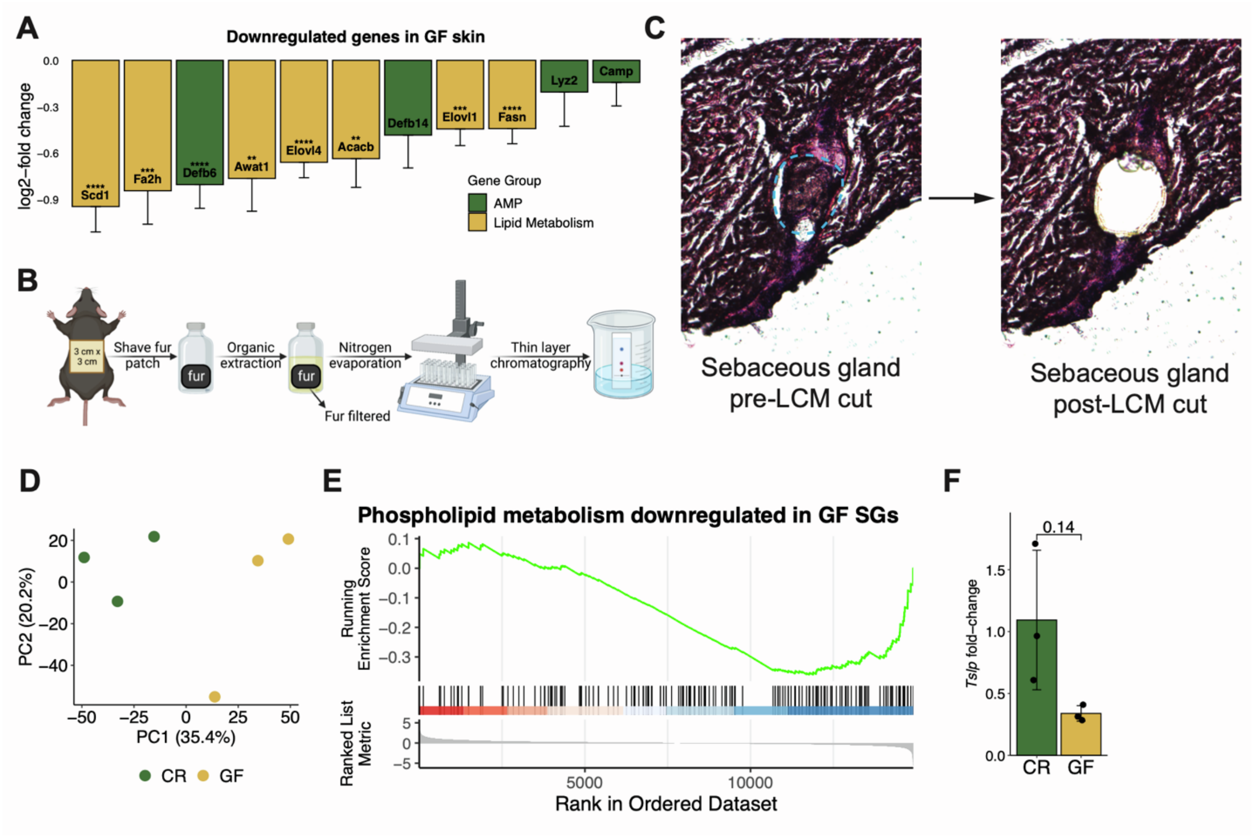

Figure S1. Skin and SG transcriptional profiles are dysregulated in GF mice, Related to Figure 1. (**A**) mRNA expression of lipid-associated and antimicrobial peptide genes in GF mice compared to CR mouse epidermis ($n = 8$ mice/group). p values are indicated on the y-axis, based on Fisher's exact test and FDR-adjusted. (**B**) Schematic detailing the thin layer chromatography method for extracting and measuring sebum secretion. (**C**) Visual representation of SG tissue isolated using LCM. (**D**) Principal component analysis showing principal component 1 (PC1) and PC2 for RNA-seq from the SG of CR or GF mouse skin ($n = 3$ mice/group). (**E**) GSEA plot displaying downregulated phospholipid pathway in the GF SG using BH-adjusted P value < 0.05. Genes shown in ranked order by running enrichment scores. (**F**) Skin mRNA expression of *Tslp* ($n = 3$ mice/group, qPCR normalized to *Gapdh* expression). No label, not significant, $^{**}p < 0.01$,

***$p < 0.001$, ****$p < 0.0001$, by Fisher’s exact test, FDR-adjusted. Data are shown as mean ± SD.

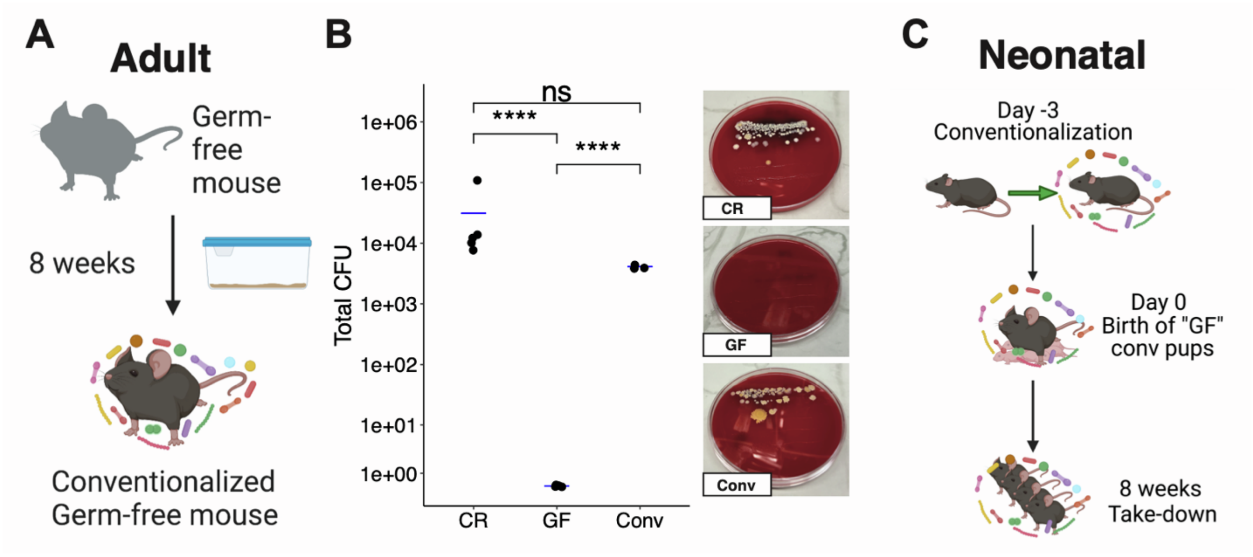

Figure S2. A similar bacterial burden in GF breeding combinations led to differential *Tslp* expression and T cell number in GF×GF F_1 skin, Related to Figure 2. (**A**) Schematic representing adult GF conventionalization techniques, whereby 8 week-old adult mice are introduced to a cage containing CR bedding and cage material for 8 weeks. (**B**) Colony forming unit (CFU) quantification from back swabs of CR, GF and conventionalized adult mice post 8 weeks of colonization. (**C**) Schematic representing neonatal GF conventionalization techniques, whereby a pregnant GF mouse is introduced to a cage containing CR bedding and cage material, giving birth to GF-derived neonatally conventionalized mice. All experiments performed 2-3 times. ns, not significant, ****$p < 0.0001$ by Student t test. Data are shown as mean ± SD.

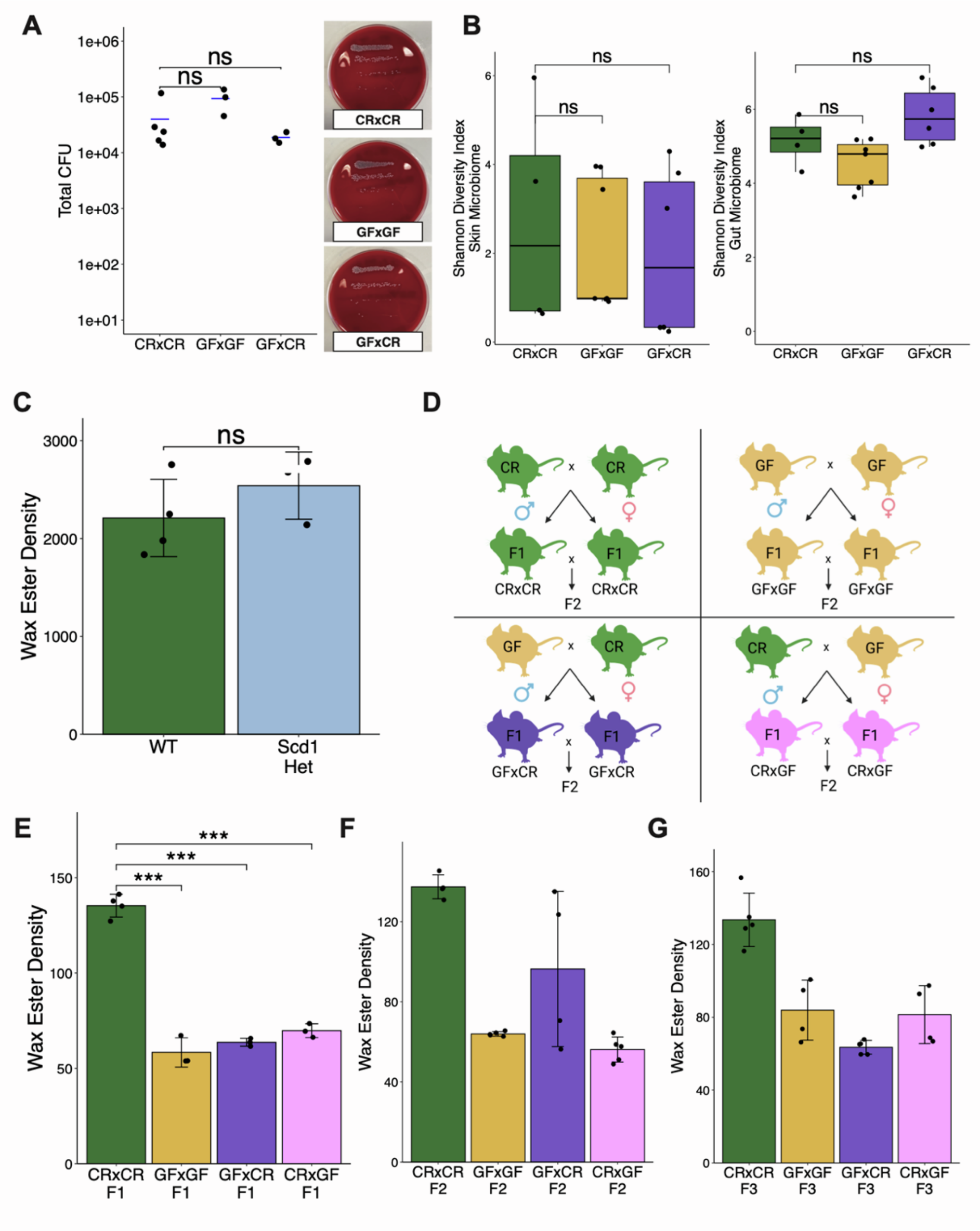

Figure S3. GF sebum defect persists to F_3 generation when using an inter-litter breeding scheme independent of microbial composition and SG defects, Related to Figure 3. (**A**) CFU quantification from back swabs of F_1 CR×CR, GF×GF, and GF×CR mice. (**B**) Alpha diversity (Shannon Diversity Index) measurement of skin and gut microbial compositions comparing F_2

CR×CR, GF×GF, and GF×CR mice (n = 4 to 7 mice/group) (**C**) TLC quantification of hair wax esters comparing WT to *Scd1*$^{+/-}$ mice (n = 3 to 4 mice/group) (**D**) Breeding scheme for alternative transgenerational experiments using littermates for multigenerational breeding. (**E to G**) TLC quantification of hair wax esters from combinatorial CR and GF natural breeding in the (E) F_1 (n = 3 to 4 mice/group), (F) F_2 (n = 4 to 5 mice/group), and (G) F_3 (n = 4 to 5 mice/group) generations. ns, not significant, $*p < 0.05$, $**p < 0.01$, $***p < 0.001$, $****p < 0.0001$ by Student t test. Data are shown as mean ± SD.

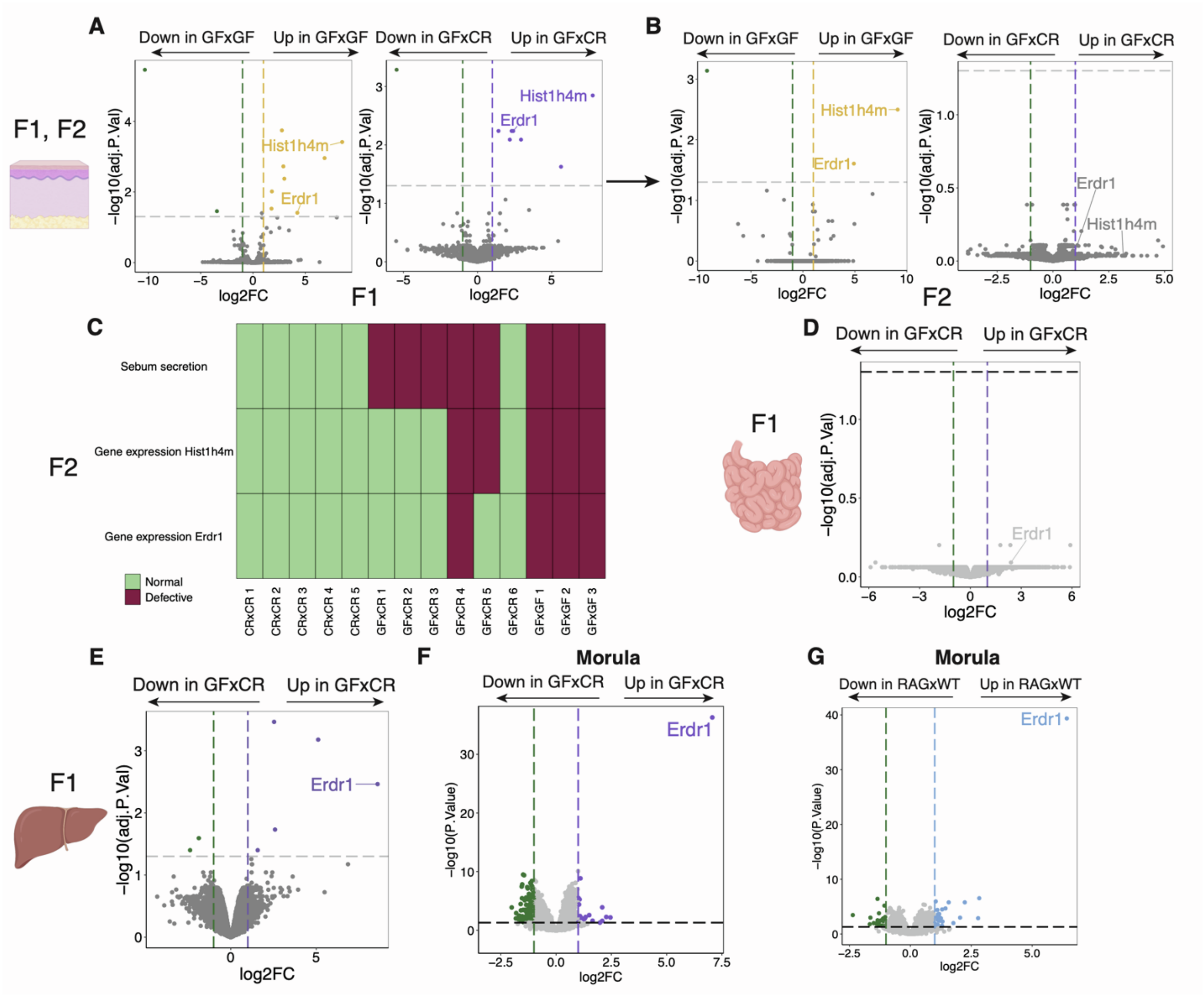

Figure S4. Effect of the absence of the microbiome or T cells on gene expression in multiple tissues in the F_1 or F_2 generations, Related to Figures 4, 5, and 6. (**A** and **B**) Gene expression by RNA-seq of F_1 and F_2 CR×CR, GF×GF, and GF×CR back skin (*n* = 3 to 6 mice/group). Volcano plots representing pairwise group comparisons of DEGs (using Benjamini-Hochberg adjusted p-value) across (A) F_1 and (B) F_2 generations, highlighting two common genes. (**C**) Heatmap comparing recovery of sebum secretion or gene expression of *Hist1h4m* and *Erdr1* in F_2 GF×CR and GF×GF mice (**D**) Gene expression by RNA-seq of F_1 CR×CR and GF×CR small intestine (*n* = 3 or 4 mice/group) portraying DEGs (using Benjamini-Hochberg adjusted p-value). (**E**) Gene expression by RNA-seq of F_1 and CR×CR and GF×CR liver tissue (*n* = 3 or 4 mice/group)

portraying DEGs (using Benjamini-Hochberg adjusted p-value). (**F**) Gene expression by RNA-seq of CR×CR and GF×CR morulae (*n* = at least 17 morulae/group, collected over three biological replicates of IVF). (**G**) Gene expression by RNA-seq of WT×WT and RAG×WT morulae (*n* = at least 28 morulae/group, collected over three biological replicates of IVF). Sequencing experiments performed once.

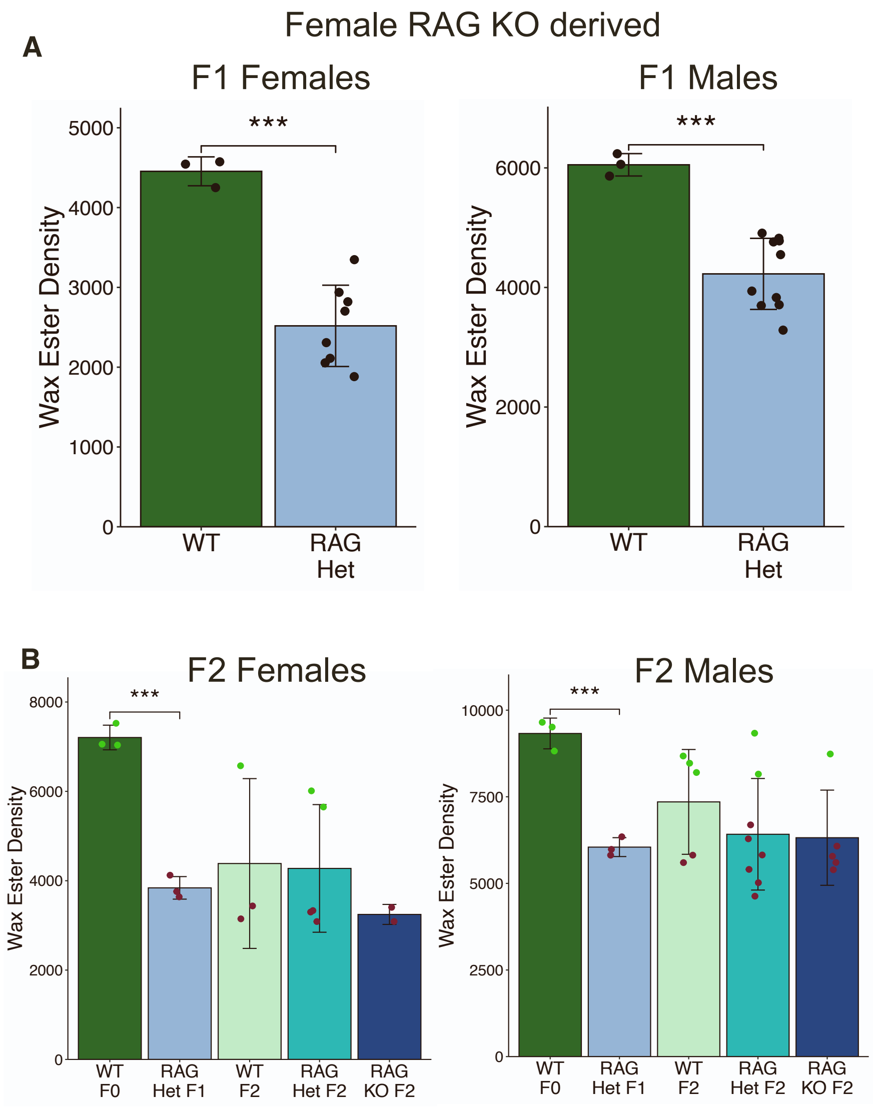

Figure S5. *Rag2*$^{-/-}$ female mice crossed with WT male mice also lead to defective *Rag2*$^{+/-}$ F_1 mice and partially rescued F_2 mice independent of genotype, Related to Figure 6. (A) TLC quantification of hair wax esters from female and male WT and F_1 *Rag2*$^{+/-}$ mice (n = 3 (WT) or 8-10 (*Rag2*$^{+/-}$) mice/group). Experiment performed twice. (**B**) TLC quantification of hair wax esters from female and male WT, F_1 *Rag2*$^{+/-}$, and F_2 WT, *Rag2*$^{+/-}$, and *Rag2*$^{-/-}$ mice (n = 2 to 8 mice/group). Point colors represent physiologic (green) or defective (red) levels of sebum secretion. ***$p < 0.001$ by Student t test. Data are shown as mean ± SD.
